# Obesogens in Prostate Cancer: An Endocrine and Metabolic Threat

**DOI:** 10.1007/s13679-026-00690-y

**Published:** 2026-02-25

**Authors:** Mariana Feijó, Lara R. S. Fonseca, Endre Kiss-Toth, Sílvia Socorro, Sara Correia

**Affiliations:** 1 RISE-Health, Department of Chemistry, Faculty of Sciences, University of Beira Interior, Covilhã, Portugal; 2 School of Medicine and Population Health, University of Sheffield, Sheffield, UK; 3 Present Address: RISE-Health, Department of Medical Sciences, Faculty of Health Sciences, University of Beira Interior, Covilhã, Portugal

**Keywords:** Adipose tissue, Endocrine-disrupting chemicals, Obesity, Obesogens, Prostate cancer

## Abstract

**Purpose of Review:**

This review addresses the contribution of obesogenic endocrine-disrupting chemicals (EDCs) to prostate carcinogenesis. It provides an in-depth overview of obesogens, tracing their mechanisms of action and effects impacting prostate cell fate. The direct effects of obesogens in disrupting adipose tissue and metabolic homeostasis, as well as disturbing prostate cells, are discussed, along with the potential indirect effects mediated by the dysregulation of the adipose tissue.

**Recent Findings:**

Obesogens represent a group of EDCs that interfere with endocrine and metabolic processes, underpinning the spread of obesity. Moreover, the ubiquitous presence in the environment, the ability to accumulate in adipose tissue and the broad range of effects targeting several biological pathways highlight that obesogens can be detrimental to human health beyond their action on promoting obesity. Prostate cancer (PCa) is a hormone-dependent cancer for which environmental influences and obesity are established risk factors, with emerging evidence suggesting that obesogens may affect its development and progression.

**Summary:**

The available data indicate that obesogens may contribute to the development of PCa. They can have direct actions in prostate cells modulating signalling pathways that drive tumour aggressiveness. Moreover, the adipose tissue dysregulated by obesogens can acquire an obesity-like phenotype, which may play a crucial role in facilitating tumour growth. Further research is needed to clarify the liaison between obesogen-induced dysregulation of the periprostatic adipose tissue depot and PCa aggressiveness. Unravelling this complex crosstalk will be pivotal for identifying novel therapeutic strategies and preventing aggressive PCa, especially in obese patients.

## Introduction

Obesity is a major global health challenge and is widely recognised as the epidemic of the twenty-first century [1]. In Europe, projections estimate that 50% of the population, including adults, young people and children, will be obese by 2030 [2, 3]. The concern with this alarming scenario has been further exacerbated in recent years, coinciding with the growing recognition that obesity is a condition influenced by factors beyond dietary habits, sedentary life or genetic predisposition [4].

Environmental factors, particularly endocrine-disrupting chemicals (EDCs), a set of environmental compounds that interfere with normal endocrine signalling by altering hormone production, bioavailability, metabolism or mechanism of action [5], have been identified as significant contributors to the spread of obesity. EDCs may affect obesity because some of these compounds have the ability of both disrupting endocrine homeostasis and inducing metabolic dysregulation, thus promoting adipogenesis, morphofunctional changes in the adipose tissue, and alterations in key regulatory pathways involved in the neural control of appetite, satiety, and energy balance [6–8]. EDCs with actions driving fat accumulation and obesity are called obesogens, among which the organotin tributyltin (TBT) is considered the “obesogen model” [9–11]. Proposed over a decade ago, the “obesogen hypothesis” as a major driver of obesity has gained increasing support with the growing list of identified EDCs with obesogenic properties [12–14].

Obesogens are widespread in the environment, reaching humans through their various types of use and distinct exposure routes. Their ubiquitous presence in the environment, namely as biocides, pesticides, plasticisers, flame retardants and additives in food, cosmetics, and pharmaceuticals, as well as their preferential accumulation in adipose tissue, their primary target, raises substantial concerns about their potential impact on human health [7]. Obesogen-induced dysregulation of lipid homeostasis primarily occurs through the activation of nuclear transcriptional regulators, such as the peroxisome proliferator-activated receptor gamma (PPAR-γ) and retinoid X receptor (RXR) heterodimer, which favours adipocyte proliferation and differentiation, and, consequently, enhances its functionality [10, 13]. Evidence also suggests that obesogens can interfere with several other biological pathways. Their ability to bind different classes of nuclear receptors, thereby disrupting endocrine signalling and governing downstream pathways related to survival and inflammation, underscores their broad impact on human health beyond obesity [13, 15–17].

Over the last years, an increasing body of research findings has been linking obesity with the development of prostate cancer (PCa) [18–20]. Strong evidence shows that obese men have a significantly higher risk of being diagnosed with aggressive, high-grade PCa than men of a healthy weight [18–20]. Obese PCa patients display accelerated tumour progression to the aggressive, castrate-resistant stages of disease, which has a very poor clinical prognosis [20].

Interestingly, it was also shown that extrinsic factors contribute to over 70% of the risk for PCa development [21], which strongly supports the existence of a link between environmental influences, namely EDCs and obesogens, and PCa. Indeed, in recent years, several studies have demonstrated that EDCs play a significant role in promoting PCa development, with comprehensive reviews further addressing and consolidating this evidence [22–25]. The ability of obesogenic EDCs to disrupt endocrine and metabolic homeostasis has highlighted that they represent a dual threat in prostate carcinogenesis, underpinned by the hormone-dependent nature of PCa and the well-known metabolic plasticity of cancer cells under environmental stress [26]. The present review provides an in-depth overview of currently identified obesogens, discusses their mechanisms of action in adipose tissue and systemic metabolism, and explores their potential direct effects on prostate cells, as well as their indirect effects mediated by the dysregulation induced in adipose tissue and its secretome. Moreover, the potential druggable targets to mitigate the effects of obesogens in PCa are also discussed**.**

## The Interaction Between Obesity and EDCs (Obesogens)

This section provides an overview of the EDCs with potential obesogenic effects identified to date, exploring the molecular mechanisms that lead to fat accumulation, and metabolic, inflammatory, and oxidative impairment.

### Adipose Tissue Dysfunction and Obesity

**Fig. 1:**
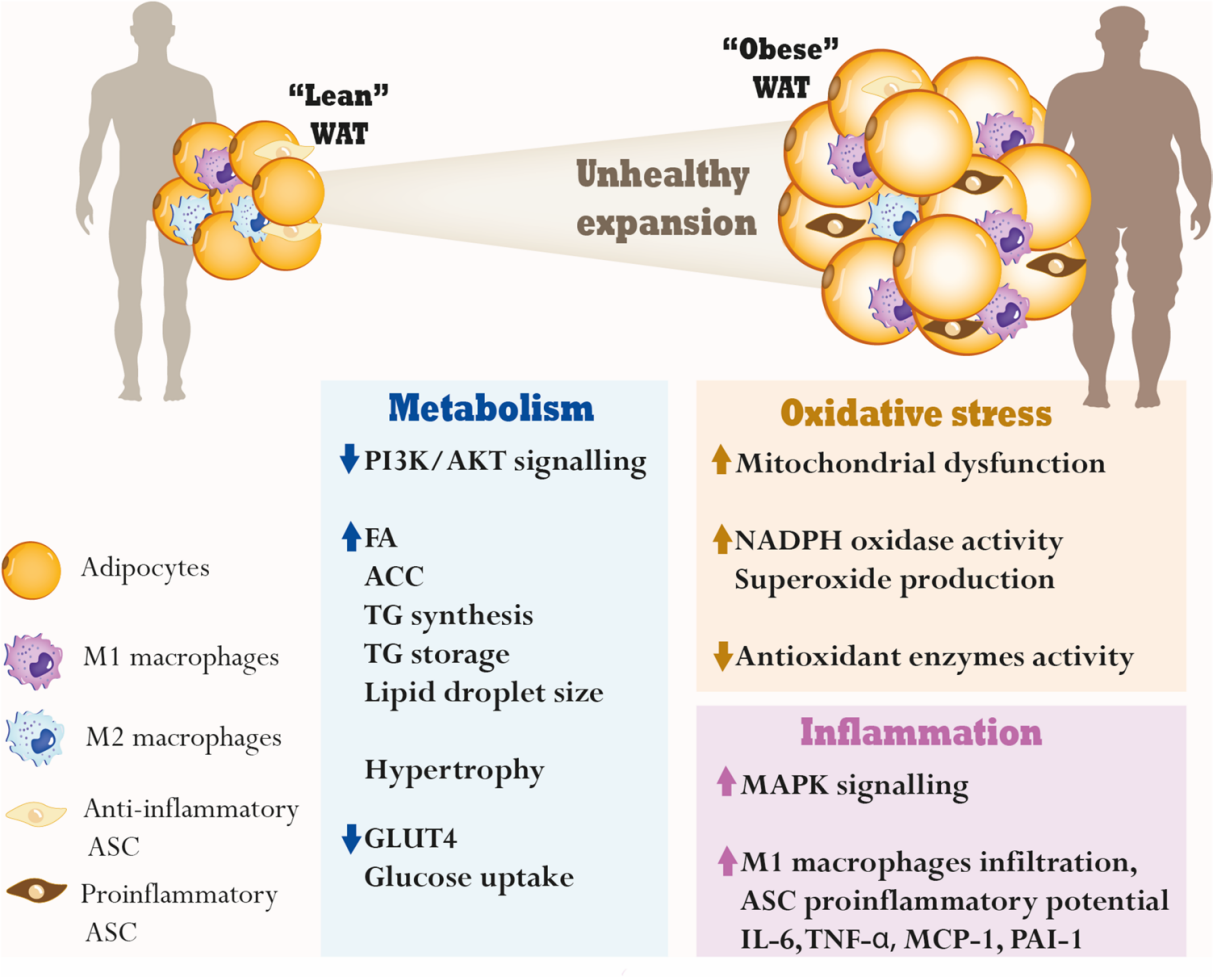
White adipose tissue (WAT) dysregulation in obesity. Unhealthy expansion of adipose tissue is observed in obesity, mostly by adipocyte hypertrophy, which results from obesity-dysregulated metabolism in the WAT, with higher fatty acid (FA) and acetyl coenzyme-A carboxylase (ACC) levels, favouring triglyceride (TG) synthesis and storage, with increased lipid droplet size. Downregulation of phosphatidylinositol 3-kinase (PI3K)/protein kinase B (AKT) signalling (crucial for metabolic homeostasis), and reduced expression of glucose transporter 4 (GLUT4) expression and glucose uptake, are also observed. Increased oxidative stress is associated with obesity, with an increased rate of adipocyte superoxide production, nicotinamide adenine dinucleotide phosphate (NADPH) oxidase activity and downregulation of antioxidant enzymes. Besides metabolic and oxidative impairment, obesity is also associated with the production of pro-inflammatory adipokines, including interleukin-6 (IL-6), tumour necrosis factor-alpha (TNF-α), monocyte chemoattractant protein 1 (MCP-1), and plasminogen activator inhibitor-1 (PAI-1), proinflammatory macrophage infiltration (M1 macrophages) and altered adipose stem cells’ (ASC) immunomodulation properties (more proinflammatory). The mitogen-activated protein kinase (MAPK) signalling pathway is also a central mediator in the development of obesity, with its activation being related to inflammation in adipose tissue. M2 macrophages: anti-inflammatory

White adipose tissue (WAT) is primarily involved in energy storage, in contrast to brown adipose tissue, which is specialised in energy dissipation through conversion of chemical energy into heat [27, 28]. WAT has a central role in maintaining metabolic homeostasis, both at the organ and systemic levels, serving as the primary reservoir for triglycerides (TGs) and regulating their mobilisation [29–31]. Therefore, WAT is the main origin of the significant metabolic alterations that underpin adipocyte dysfunction and obesity Fig. 1. WAT is mainly composed of large, lipid-storing white adipocytes, but also contains a complex stromal vascular fraction (SVF) with various cells like preadipocytes, fibroblasts, endothelial cells, immune cells (mainly macrophages), and mesenchymal stem cells (MSCs) [32]. Adipose stem cells (ASCs), a mesenchymal stem cell subset, are important components of the adipose SVF and exhibit multilineage differentiation (adipogenic, osteogenic, chondrogenic) and self-renewal [33]. Beyond their role in adipose tissue development and homeostasis, ASCs have been implicated in the pathogenesis of obesity and obesity-related metabolic disorders through altered immunomodulation properties Fig. 1 [34, 35]. In turn, obesity-associated adipocyte dysfunction alters ASC abundance and function, contributing to impaired adipose tissue remodelling and increased metabolic disease risk [36, 37].

WAT is distributed throughout the body, being mainly classified as subcutaneous (sWAT) and visceral (vWAT), but also including inter-/intramuscular and bone marrow depots [38]. Among the WAT types, the sWAT and vWAT are of the most metabolic importance [38]. The main depots of sWAT are abdominal, subscapular, gluteal and thigh, while vWAT is distributed inside the peritoneum and around internal organs, protecting them from physical damage [38]. Increased vWAT accumulation is positively associated with the onset of metabolic diseases, whereas sWAT shows little association [39, 40].

Under conditions of energy surplus, adipocytes synthesise TGs from free fatty acids released from circulating TG-rich lipoproteins, such as chylomicrons and very-low-density lipoproteins via the action of lipoprotein lipase (LPL) [41]. High glucose levels and their metabolization, with the consequent elevated production of acetyl-CoA, drive the de novo lipogenesis, increasing levels of fatty acids that are sequentially esterified to glycerol, resulting in the formation of TGs [41]. Prolonged excessive storage of TG, as occurs in obesity, leads to an increase in the size of lipid droplets, resulting in the expansion of adipose tissue [41]. Two cellular mechanisms are responsible for adipose tissue expansion: i) hypertrophy, characterised by enhanced TG storage as previously mentioned, and ii) hyperplasia, which results from the increase in adipocyte cell number by enhanced rates of adipogenesis, *i.e.* the formation of new preadipocytes from stem cells [42].

Adipogenesis involves multiple steps, including the activation of specific adipogenic transcription factors, including peroxisome PPAR-γ, CCAAT/enhancer-binding proteins (C/EBPs), and sterol regulatory element binding protein (SREBP), which shape the mRNA/protein networks driving adipocyte development [43–45]. Expansion of adipose tissue and obesity have been categorised into two types: i) metabolically healthy, with preferred lipid storage in sWAT, and ii) metabolically unhealthy, with impaired expandability of sWAT resulting in ectopic fat accumulation and excessive vWAT [46]. As vWAT contacts with the internal organs and shares their vasculature, metabolites and adipokines (*e.g.*, leptin, adiponectin, and resistin) secreted from its adipocytes rapidly reach the adjacent organs [46], altering their physiological responses. Nevertheless, a metabolomic study revealed that the “obese” WAT exhibits marked changes in lipid, amino acid, carbohydrate and nucleotide metabolism [32]. Higher levels of several fatty acids and lipid metabolites belonging to the linoleic acid metabolism subfamily were observed, with elevated abundance of arachidonic acid as well as metabolites involved in arachidonic acid synthesis, including linolenic and dihomo-linolenic acids [32]. Tissue levels of succinate and malate were also elevated, and metabolites that could enter the Krebs cycle via anaplerosis were mostly diminished, suggesting the existence of mitochondrial dysfunction [32]. Moreover, the authors proposed a relationship between the verified trend towards an increased rate of non-mitochondrial oxygen consumption and enhanced adipocyte superoxide production due to cytosolic oxidase activity [32, 47]. This possibility is consistent with other studies showing increased oxidative stress (OS) in the adipocytes in obesity, predominantly caused by an increase in NADPH oxidase activity and downregulation of antioxidant enzymes [48–50]. With respect to glycolysis in obese adipocytes, a decrease in glucose 6-phosphate accompanied by increased intracellular glucose levels was observed, which implied hexokinase as a possible major rate-limiting step in the provision of glucose carbons required for TG synthesis [32]. The expression of glucose transporter 4 (GLUT4), which plays a pivotal role in glucose uptake in adipose tissue, was shown to be reduced in obese individuals [51]. However, the reduction in GLUT4 expression is more strongly correlated with insulin resistance and diabetes than with obesity alone [51].

Globally, the dysregulation of adipose tissue Fig. 1 and the alterations in “lean” vs. “obese” WAT result in extensive tissue remodelling and local and systemic metabolic dysfunction, which contributes to the development of obesity-associated comorbidities, such as insulin resistance and diabetes [42]. Obesity is also a significant risk factor for other diseases, such as cardiovascular disorders, respiratory and skin diseases, and cancer [52–55].

The phosphatidylinositol 3-kinase (PI3K)/protein kinase B (AKT), adenosine monophosphate-activated protein kinase (AMPK) and the mitogen-activated protein kinase (MAPK) signalling pathways are relevant regulatory mechanisms of adipose tissue function, which are linked to obesity. PI3K/AKT signalling, primarily activated by insulin, is crucial for metabolic homeostasis, regulating energy storage and lipid synthesis and breakdown, as well as controlling glucose uptake into adipocytes and other insulin-sensitive tissues, including the promotion of adipogenesis and adipocyte differentiation [56, 57]. Accordingly, the dysregulation of this signalling pathway contributes to the development of obesity and the chronic inflammation and metabolic dysregulation in adipose tissue [56, 57]. Under conditions of excessive energy intake, the PI3K/AKT pathway is suppressed in adipocytes, leading to increased lipolysis and decreased glucose uptake, elevating circulating fatty acids and leading to chronic inflammation, ectopic lipid accumulation and glucose metabolism imbalance [57].

AMPK is a central regulator of cellular and systemic energy balance. AMPK activation by phosphorylation was shown to be protective in obesity [58, 59]. AMPK activation inhibits adipogenesis and lipogenesis through inactivation of PPAR-γ, C/EBPα, acetyl-CoA carboxylase (ACC), fatty acid synthesis products, and SREBP-1c in adipose tissue [58, 59]. AMPK activation was also shown to increase thermogenesis and energy expenditure in the WAT of mice fed an HFD [60].

The MAPK signalling pathway, which includes ERK1/2, c-Jun N-terminal kinase, and p38 MAPK, is also a central mediator in the development of obesity [61–64]. The activation of MAPKs is related to adipocyte hyperplasia and inflammatory cell infiltration [64]. Indeed, besides metabolic dysfunction, obesity is associated with the production of inflammation-related adipokines and the population of proinflammatory macrophages in adipose tissue, leading to chronic low-grade inflammation [65]. Remarkably, proinflammatory cytokines often increased in obesity, including those from the interleukin (IL) cytokine family (*e.g.* IL-6), interferon γ, tumour necrosis factor-α (TNF-α), monocyte chemoattractant protein 1 (MCP-1), and plasminogen activator inhibitor 1, are more abundant in vWAT than in sWAT [66, 67].

"Obese” adipose tissue, presenting excessive adiposity, impaired metabolic function and an inflammatory status, by its communication with distant organs via blood stream circulation or by the possibility of paracrine actions in adjacent tissues, might play a crucial role in disrupting cell fate and promoting cancer development.

### The Obesogen Model Tributyltin and Other Organotins

Organotins are tetravalent tin-based compounds characterised by mono-, di-, tri-, or tetra-substituted organic functional groups [68]. Many applications have been found for these chemicals in industrial processes and as broad-spectrum biocides, with hundreds of derivatives routinely employed [68].

TBT, the prototypical obesogen, is a triorganotin compound extensively used in antifouling paint formulations due to its biocidal properties [11]. Additional applications include cooling systems, wood pulping, leather processing, wood preservation, and textile treatments [69]. The global peak usage of TBT occurred in the 1980 s, being officially banned worldwide in 2008 [70]. Nevertheless, several “TBT hotspots” persist nowadays in various European coastal areas, in countries like England, Spain, and Italy [71–78]. Moreover, Uc-Peraza et al. reported that an extensive list of TBT-based antifouling products was available in the market of several countries in 2021 [79].

TBT is highly persistent in aquatic environments, particularly accumulating in sediments with elevated organic content or low/non-existent oxygenation (hypoxic/anoxic conditions) [80, 81]. Its half-life can range from 1 to 5 years in well-oxygenated marine sediments but may extend to several decades in O_2_-deprived soils [80, 81]. Furthermore, TBT slowly leaches from sediments back into the seawater, representing a long-term source for recontamination [80, 81].

Triphenyltin (TPT) is another organotin previously used as a fungicide and miticide in the 1950 s, which shares similar toxicological properties with TBT and has likewise been banned due to its persistence and bioaccumulation potential [82].

Human exposure to TBT and other organotins primarily occurs through the consumption of contaminated seafood. Over the last decade, several studies demonstrated that exposure to TBT through the diet is a matter of concern. Significant levels of TBT were detected in edible gastropods (602.3 ± 14.5 ng Sn/g), crustaceans and molluscs (0.19 µg/kg), commercial oysters (six different seafood markets, 68.1 ± 20.1 ng Sn/g), fish (0.32 µg/kg), blended (28.8 ± 2.82 µg/kg)/sunflower seed (26.9 ± 2.15 µg/kg)/soybean (13.1 ± 1.24 µg/kg) oils and even in random duplicate diet samples (TBT and its metabolites detected in 7 out of 28 samples) [83–87].

**Fig. 2:**
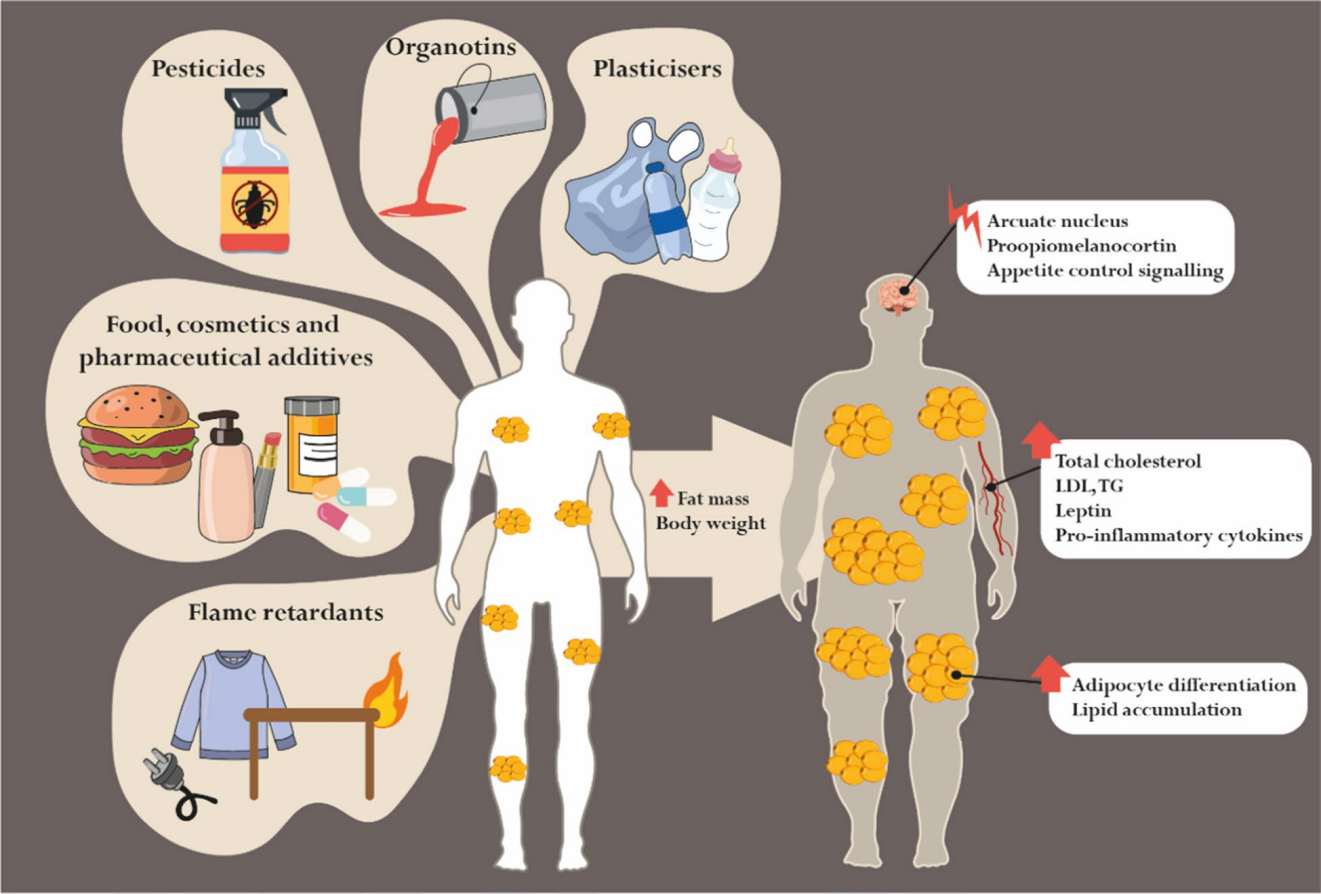
Obesogens: sources and biological effects. Endocrine-disrupting chemicals with obesogenic potential are ubiquitous in human daily life activities, being present in a wide range of products, including food, cosmetics, pharmaceuticals, anti-fouling paints (organotins), pesticides, flame retardants, plastics, clothing, furniture, and electronics, among others. These compounds dysregulate lipid metabolism and stimulate adipogenesis, leading to increased fat mass and body weight. In addition to morphological and functional changes in adipose tissue, obesogens can also increase circulating levels of leptin and pro-inflammatory cytokines, and affect key regulatory pathways involved in the neural control of appetite, satiety, and energy balance, namely the arcuate nucleus response and proopiomelanocortin secretion. LDL: low-density lipoprotein; TG: triglycerides

The potential of TBT and other organotins to act as obesogens and to interfere with the endocrine regulation of adipogenesis is well recognised Table 1, Fig. 2. Both TBT and TPT have been demonstrated to act as high-affinity nanomolar ligands for the master regulator of adipogenesis, the PPAR-γ-RXR heterodimer, stimulating differentiation of preadipocytes into adipocytes [9, 10, 88]. Exploring PPAR-γ-RXR agonism in the murine 3T3-L1 preadipocyte cell line model, various studies confirm organotins as effective promoters of adipocyte differentiation, even at nanomolar concentrations [10, 11, 88, 89]. In general, these compounds stimulate preadipocyte maturation, increasing lipid droplets and the expression of terminal differentiation markers, such as fatty acid-binding protein 4 (FABP4), with decreased expression of GLUT4 [10, 11, 88–90]. Moreover, both TBT and TPT seem to be capable of modulating the expression of lipid metabolism-related genes in the liver, as shown in rainbow trout (RTL-W1) and human (HepaRG and HepG2) liver cells [91, 92]. In this context, a recent study demonstrated that the transcriptional changes induced by TBT in the liver of male mice are more pronounced than those in the female group, with significant enrichment of genes involved in cellular ketone metabolism and fatty acid metabolism [93]. Studies using in vivo models have further elucidated the systemic effects of TBT and other organotins in lipid handling and adipogenesis. Peripubertal exposure to low doses of TBT (0.5–50 µg/kg) in mice has been associated with increased body weight gain [94, 95]. Interestingly, low-dose exposures seem to exert more pronounced obesogenic effects. In pubertal male rats, a 30-day exposure to TBT or TPT (0.5–15 and 2–12 mg/kg, respectively) led to significant weight gain only in animals receiving the lowest dose of TPT (2 mg/kg) [96]. Besides dose-dependent effects, Penza et al. hypothesised that the obesogenic potential of TBT (0.05–500 µg/kg) could be sex-dependent, as proved by differential adipose and body weight changes, where male mice rapidly responded to low-dose TBT, translated by increased fat/body weight ratio and changed adipose tissue architecture [97], which may be related with the different androgenic milieu in male and female, as androgens are target regulators of lipid handling [98]. Androgens modulate lipid metabolism through complex mechanisms that involve direct gene regulation and the indirect modulation of transcription factors, as seen in the activation of sterol regulatory element-binding proteins [99–102]. This activation leads to coordinated upregulation of enzymes involved in lipid synthesis, contributing to increased lipogenesis [99–101]. These hormones can also modulate the activity of other transcription factors involved in lipogenesis and lipid metabolism, such as PPAR-γ [101]. Indeed, TBT’s actions have been described as potentially androgenic, capable of promoting androgen-dependent transcription and proliferation [15, 103]. Transgenerational effects of TBT were also observed. Prenatal exposure (5.42, 54.2, or 542 nM) for 7 days before mating increased WAT depot weight, adipocyte size, and adipocyte number, as well as reprogrammed MSCs toward the adipocyte lineage in the following three generations [104]. Overall, existing studies highlight the potential of environmental exposure to triorganotins and their effect in disturbing lipid homeostasis. This disruption may alter adipocyte physiology and the flow of nutrient reserves between growth and storage processes, ultimately resulting in increased adipose tissue mass.

**Table 1:** Endocrine-disrupting chemicals (EDCs) with potential obesogenic effects: classes, sources and reported actions

Class	EDC	Source	Obesogenic actions	Refs
Pesticides	TBT (organotin, biocide)	Cooling systems, wood pulping, leather processing, wood preservation and textile treatments	↑ BW gain and fat mass/BW ratio Changed adipose tissue architecture ↑ Adipocyte lipid content ↑ Lipid accumulation in adipose depots, liver, and testis ↑ Adipocyte differentiation (PPAR-γ/RXRα-dependent) ↑ Expression of FABP4, FASN, LPL, and ABCA1	[1–9]
	TPT (organotin, fungicide, miticide)	Food and ornamental crops	↑ BW gain ↑ Adipocyte differentiation (PPAR-γ/RXR-dependent)	[1, 8]
	DDT (insecticide)	Insect-borne diseases Food and ornamental crops, and livestock	Positively associated with BMI, waist circumference and F2 and F3 obesity ↑ Risk of becoming overweight/obese (only in males)	[10–15]
	DDE (DDT metabolite)	-	↑ Risk of becoming overweight or obese	[12–14]
	Glyphosate (insecticide)	Weeds, silviculture, domestic gardens, and urban areas	↑ HFD-induced fat accumulation, BW gain, and inflammation ↑ Obesity frequency in F2 and F3 ↑ TG plasma levels	[16–19]
	Malathion (insecticide)	Food and ornamental crops, livestock, and head/body lice	↑ TG and LDL plasma levels	[20–22]
	Chlorpyrifos (insecticide)	Food and ornamental crops	↑ BW gain (more accentuated in males) ↑ Adipocyte differentiation and lipid droplet storage capability ↑ Expression of C/EBPα, PPAR-γ and FABP4 ↑ TG and LDL plasma levels ↓ HDL plasma levels	[23–27]
	Diazinon (insecticide)	Food crops and livestock	↑ Expression of PPAR-γ, FASN, ACC, FABP4, LPL, adiponectin and perilipin ↑ TG and LDL plasma levels ↓ HDL and phospholipid plasma levels	[28–30]
	Dichlorvos (insecticide)	Ornamental crops, livestock and anthelmintic applications	Lipid metabolism dysregulation	[31, 32]
	Imidacloprid (insecticide)	Food and ornamental crops, pet care and household pests	Positively associated with waist circumference, overweight and obesity ↑ Adipocyte differentiation and lipid accumulation, via AMPKα signalling	[33–37]
	Triflumizole (fungicide)	Food and ornamental crops	↑ Adipocyte differentiation (PPAR-γ-dependent) ↑ Adipose depot weight ↑ Expression of FABP4 and PPAR-γ	[38]
	Quizalofop-p-ethyl (herbicide)	Food and ornamental crops	↑ TG accumulation (partially dependent on PPAR-γ)	[39]
Plasticisers	BPA	Polycarbonate plastics and epoxy resins, *e.g.* feeding bottles, plastic food containers and thermal paper	↑ BW gain ↑ Expression and activity of 11β-HSD1 ↑ Lipid accumulation and expression of PPAR-γ, FABP4, CD36 and LPL in adipocytes ↑ Expression of PPAR-γ, C/EBPα and GLUT4 during foetal adipogenic differentiation ↑ Pro-inflammatory cytokine release by mature adipocytes, specifically CCL20, IL-18, IL-6, IL-1β and TNF-α ↑ Adipose tissue macrophages' self-renewal and M1 polarisation	[5, 40–45]
	BPS (BPA substitute)	Hard plastics, synthetic fibres for clothing, thermal paper, and epoxy resins	↑ Overweight, fat mass and hyperleptinemia in HFD-fed male offspring ↑ Adipogenesis in subcutaneous and visceral preadipocytes ↑ Expression of IL-6, IL-8 and IL-1β in visceral adipocytes	[46–49]
	DEHP	Flexible plastics, various consumer and industrial products, including medical devices, food packaging, shower curtains, vinyl upholstery, floor tiles, garden hoses, among others	↑ BW gain and fat mass weight Positively associated with BMI and waist circumference ↑ Adipocyte proliferation ↑ Expression of PPAR-γ and C/EBPα in adipocytes ↑ Adipocyte secretion of IL-8 and MCP1	[50–56]
	MEHP (DEHP metabolite)	-	↑ Adipocyte proliferation and differentiation (PPAR-γ-dependent) ↑ Expression of PPAR-γ, AP2 and LPL in peri-epididymal adipose tissue ↑ Cholesterol, TG and glucose serum levels	[57–59]
Food, cosmetics and pharmaceutical additives	Carboxymethylcellulose (emulsifier)	Food, eye drops and lubricants, cosmetics	↑ BW and white adipose tissue depot weight	[60]
	P-80 (emulsifier)	Food, cosmetics, vaccines, drug excipient	↑ BW and white adipose tissue depot weight	[60, 61]
	DOSS (surfactant)	Food, cleaning and furnishing care products, pharmaceutical formulations	↑ BW and visceral adipose tissue weight (male offspring) ↑ Adipocyte differentiation (PPAR-γ-dependent) ↑ Leptin and IL-6 plasma levels (male offspring) ↓ Adiponectin plasma levels (male offspring)	[62, 63]
	Span-80 (Surfactant)	Food, cosmetics, and pharmaceuticals	↑ Adipocyte differentiation (RXRα-dependent)	[64]
	3-BHA (Preservative)	Food, food packaging, cosmetics	↑ Obesogenic effects with HFD ↑ BW, white adipose tissue weight and adipocyte enlargement ↑ Adipocyte proliferation and differentiation ↑ Expression of PPAR-γ, C/EBPα, FABP4, CD36, ACC, TNF-α and IL-6 in adipocytes and white adipose tissue Shift MSC differentiation into adipocytes from a brown to white-like phenotype, promoting lipogenesis ↑ TG plasma levels	[65–67]
	MSG (Flavour enhancer)	Food	Obesity inducer Impairment of glucagon-like peptide-1 secretion Damage to the hypothalamic nuclei arcuate	[68–71]
	Parabens (Preservatives)	Food, cosmetics and pharmaceuticals	Pre-natal exposure associated with child overweight ↑ Adipocyte differentiation in a PPAR-γ-dependent manner ↑ Lipid accumulation and expression of PPAR-γ, C/EBPα, FASN, FABP4, perilipin and adiponectin in adipocytes	[72–76]
	NP (surfactant)	Personal care products, detergents, industrial applications	↑ BW and fat mass ↑ Adipocyte differentiation ↑ PPAR-γ expression and TG accumulation in adipocytes ↑ 11β-HSD1 in adipose tissue ↑ Total cholesterol and serum glucose levels	[77–79]
Flame retardants	PBDEs	Electronics, furniture, toys, and foodstuffs	Accumulation in adipose tissue associated with insulin resistance in obesity ↑ Risk of obesity ↑ Visceral fat mass ↑ Obesogenic effects with HFD ↑ Expression of FABP4, perilipin, leptin C/EBPα and PPAR-γ in adipocytes ↑ TG in adipocytes ↑ Lipolysis and glucose oxidation	[80–87]
	OPFRs	Household products, electronics, furniture, textiles	Positively associated with BMI and obesity ↑ BW gain only in HFD males ↑ Adipocyte differentiation and lipid accumulation ↓ Fatty acid β-oxidation ↑ OS and expression of proinflammatory cytokines IL-1β, IL-22 ↑ Circulating leptin in HFD females	[88–93]

*↑* Increased/stimulated, *↓* Decreased, *3-BHA* 3-tert-butyl-4-hydroxyanisole, *11β-HSD1* 11β-hydroxysteroid dehydrogenase type 1, *ABCA1* Member 1 of human transporter sub-family, *ACC* Acetyl CoA carboxylase, *AMPKα* AMP-activated protein kinase alpha subunit, *AP2* Adipocyte-specific fatty acid binding protein, *BMI* Body mass index, *BPA* Bisphenol A, *BPS* Bisphenol S, *BW* Body weight, *C/EBPα* CCAAT/enhancer-binding protein alpha, *CCL* C–C Motif chemokine ligand, *DDE* Dichlorodiphenylethylene, *DDT* Dichlorodiphenyltrichloroethane, *DEHP* Di(2-ethylhexyl) phthalate, *DOSS* Dioctyl sodium sulfosuccinate, *ED* Embryonic day, *FABP4* Fatty acid-binding protein 4, *FASN* Fatty acid synthase, *FATP1* Fatty acid transport protein 1, *HDL* High-density lipoprotein, *HFD* High fat diet, *IL* Interleukin, *LDL* Low-density lipoprotein, *LPL* Lipoprotein lipase, *MCP1* Monocyte chemoattractant protein-1, *MEHP* Mono(2-ethylhexyl) phthalate, *MSC* Mesenchymal stem cells, *MSG* Monosodium glutamate, *NP* Nonylphenol, *OPFRs* Organophosphate flame retardants, *P-80* Polysorbate-80, *PBDEs* Polybrominated diphenyl ethers, *PND* Postnatal day, *PPAR-γ* Peroxisome proliferator-activated receptor gamma, *OS* Oxidative stress, *TBT* Tributyltin, *TG* Triglyceride, *TNF-α* Tumour necrosis factor alpha, *TPT* Triphenyltin.

### Pesticides

Pesticides represent a heterogeneous group of chemical compounds used extensively in agriculture, gardens, plantations, domestic contexts and other settings to control pests [105]. They are classified based on the target organism, being so-called insecticides, herbicides, rodenticides, and fungicides [105]. This panoply of applications results in widespread human exposure to these compounds, which can occur through distinct routes [105]. Most pesticides are persistent organic pollutants, characterised by resistance to degradation, high potential for biomagnification and bioaccumulation [106]. Several of these compounds have been identified as EDCs for a long time, but their ability to promote fat accumulation has only been exploited in recent years [106–108]. Pesticides can act as obesogens by increasing adipogenesis and lipid accumulation and by inducing hormonal changes that alter the regulatory pathways of appetite, satiety, and energy balance Table 1, Fig. 2 [106–108]. Importantly, it has been reported that the effects can be exacerbated when the exposed individuals are already obese [7].

Structurally, pesticides can be classified as organochlorines (OCs), organophosphates (OPs), carbamates, or pyrethroids, as well as neonicotinoids, a relatively newer class of insecticides [109, 110].

Strong evidence exists concerning the obesogenic potential of OCs and OPs. OCs are lipophilic organic compounds with chlorine atoms in their structure, making them prone to accumulate throughout the trophic chain, mainly in the adipose tissue due to their hydrophobicity [110]. One of the most studied OCs is dichlorodiphenyltrichloroethane (DDT). DDT, the first synthetic pesticide discovered, was widely used as an insecticide against malaria and typhus until it was banned in 1972 due to its deleterious impact on wildlife and human health via the food chain [111]. Exposure to DDT was associated with obesity in children and adults, and the DDT persistent metabolites, 1,1-dichloro-2,2-bis(p-chlorophenyl)ethylene (DDE) and 1,1-dichloro-2,2-bis(p-chlorophenyl)ethane (DDD), were also related to metabolic dysfunction [112–116]. Prenatal exposure to DDT and DDE increased the risk of obesity during childhood and adulthood, with existing evidence pointing to the sex-specific effects of these compounds [117–121]. DDT exposure was linked to overweight status in boys, whereas DDE exhibited a greater impact in girls [117–121]. Moreover, transgenerational obesogenic effects have also been attributed to DDT, which, worryingly, demonstrates the repercussions these compounds have on human health even long after the abolishment of their usage [122]. In a cohort study, perinatal exposure to DDT was correlated with adult obesity in the F2 generation [122]. Interestingly, in another study, although F1 generation offspring did not develop obesity, both male and female F3 generation were obese and presented differential DNA methylation in genes associated with this comorbidity [123]. A systematic review with meta-analysis presented by Cano-Sancho and co-authors aimed to classify DDT and DDE as obesogenic compounds in humans, based on a moderate level of confidence from *in vivo, in vitro* and human primary and supporting evidence [113]. The epidemiological data revealed a positive association between exposure to DDE and BMI, with exposure to DDT being positively associated with increased adiposity in rodents, supporting the biological plausibility of the obesogenic effects of the studied compounds [113]. DDT (1 μM) was shown to increase MSCs' proliferation, enhancing their differentiation despite the diminished self-renewal capacity [124]. Moreover, increased mRNA levels of several key regulators of adipogenesis and osteogenesis were observed [124]. OPs, distinguished by a central phosphorus atom bonded to oxygen or sulfur, account for approximately 40% of global pesticides used worldwide [125]. Glyphosate and malathion are among the OPs most commonly used in agricultural activities. Glyphosate is an herbicide widely employed against perennial and annual weeds, as well as in silviculture, domestic gardens, and urban areas [126]. This herbicide is considered safer than others, but its overuse in broad herbicidal activities and the development of transgenic glyphosate-resistant crops impose chronic effects on the environment and humans [127, 128]. Glyphosate accumulates both in terrestrial and aquatic environments, being absorbed by soil particles and usually detected in surface water, groundwater and sediments [129, 130]. While many studies do not assess glyphosate’s obesogenicity per se, the capacity of this compound to exacerbate the effects of a high-fat diet (HFD) is well documented, enhancing inflammation, fat accumulation, and weight gain [131, 132]. The view of glyphosate as an obesogen is also strongly supported by the fact that obesity was reported as one of the glyphosate-induced transgenerational pathologies with a significant impact on F2 and F3 generations regardless of sex [133]. Moreover, 2 year-chronic exposure to low doses (0.05 μg/L, 4 ng/kg/day) of glyphosate-based herbicide (Roundup) in drinking water resulted in higher TG levels after the first year, with a progressive increase [134]. Through a multiomics approach, this study also revealed lipotoxic effects and increased OS induced by glyphosate [134]. At higher concentrations (4–36 μg/mL), Roundup was associated with mild toxic effects in ASCs, impacting cell viability, differentiation, and alkaline phosphatase activity [135, 136].

Malathion is another OP used against a variety of insects of fruits and vegetables, mosquitoes, flies, household insects, animal parasites (ectoparasites), and head and body lice [137]. Despite being absorbed mainly by dermal contact, exposure to malathion can occur by practically all routes, including the gastrointestinal tract, mucous membranes, and lungs [137]. Studies in rodents have shown that malathion (100–400 mg/kg/day) alters lipid metabolism, increasing the plasma levels of cholesterol, TGs and low-density lipoproteins (LDL) [138–140]. Similar effects have been described for other OPs, which are also known to induce dyslipidaemia. It is the case of chlorpyrifos (discontinued in 2001, though still in use), diazinon (banned in the US and EU in 2004 and 2007, respectively) and dichlorvos (still in use, except in the EU) [141–147]. Chlorpyrifos is related to increased body weight, which is more accentuated in male offspring, and increased adipocyte differentiation [148–151]. Chlorpyrifos and, to a lesser extent, its metabolite 3,5,6-trichloropyridinol (TCP), had a metabolic influence over adipogenesis, fostering the number of differentiated 3T3-L1 preadipocytes and enhancing the storage capability of lipid droplets [151]. These effects seem to occur through the upregulation of the transcription factors C/EBPα and PPAR-γ, being related to a significantly higher expression of FABP4 [151]. Upregulation of PPAR-γ was also observed in diazinon-exposed (1–100 µM) 3T3-L1 preadipocytes, underpinned by the increased expression of fatty acid synthase (FASN), ACC, FABP4, LPL, adiponectin and perilipin, in a concentration- and time-dependent manner [152].

Concerning neonicotinoids, the first commercial compound of this group, imidacloprid, was introduced in the 1990 s, followed by others, such as thiacloprid, clothianidin, acetamiprid and sulfoxaflor [153]. Neonicotinoid pesticides are related to harmful effects on wildlife and humans, with their actions being mainly mediated by nicotinic acetylcholine receptors [109]. As a relatively new class of insecticides, studies that focus on the metabolic disruption induced by neonicotinoids are very scarce. Imidacloprid, the most studied neonicotinoid, induces lipid accumulation and adipogenesis in adipocyte cell lines and insulin resistance in male mice, in part by impairing AMPKα signalling [154–157]. Worryingly, a study performed on 7-year-old children showed a positive association between waist circumference and urinary levels of imidacloprid, as well as other neonicotinoids, with higher imidacloprid levels being associated with overweight and obesity [158].

Triflumizole, an imidazole fungicide used on many food and ornamental crops, particularly green leafy vegetables [159], was demonstrated to induce adipogenesis in 3T3-L1 preadipocytes, promoting adipogenic gene expression at low nanomolar concentrations [160]. The effects of triflumizole were blocked by the antagonization of PPAR-γ, showing its dependence on this signalling pathway [160]. When administered during gestation at approximately 400-fold below the established no observed adverse effect level (NOAEL), triflumizole increased adipose depot weight and adipogenic gene expression [160].

Interestingly, other pesticides, such as quizalofop-p-ethyl, exert their biocide effects exactly by disrupting lipid metabolism. Quizalofop-p-ethyl is a widely used aryloxyphenoxypropionate herbicide that kills weeds by inhibiting the activity of ACC in the biosynthesis of fatty acids [161]. This herbicide (5–100 µM) can cause concentration-dependent TG accumulation, relying partially on the PPAR-γ-mediated pathway [108].

Collectively, the available literature provides compelling evidence that several widely used pesticides exhibit obesogenic potential, disrupting lipid metabolism, modulating endocrine signalling, and influencing appetite regulation, which also includes transgenerational effects affecting the metabolic programming sometimes in more than one generation.

### Plasticisers

The most well-known plasticiser with endocrine-disrupting potential is bisphenol A (BPA), which is classified as an estrogenic EDC [162]. Despite the widespread restriction on its use, BPA remains present in numerous everyday products, including feeding bottles, plastic food containers and thermal paper [163–165]. Due to its environmental ubiquity, BPA has been detected in surface waters and sediments, with levels showing a continuous upward trend [162, 166–168]. The restriction and prohibition of BPA in manufacturing procedures resulted in the development of several BPA analogues, such as bisphenol AF (BPAF), Bisphenol F (BPF) and Bisphenol S (BPS) [169, 170]. However, the structural similarity of these substitutes to BPA raises concerns about comparable or even enhanced endocrine-disrupting potential, particularly given their affinity for oestrogen receptors (ERα, ERβ) [166, 169–171].

The liaison between BPA and obesity has been explored over the years Table 1, Fig. 2. BPA significantly increased the intracellular accumulation of TGs and modulated the expression of genes involved in lipid metabolism, upregulating fatty acid transport protein 1 (*fatp1*) and *fasn* in RTL-W1 trout liver cells [91]. In human visceral fat biopsies from children, low concentration BPA (10 nM), likely by glucocorticoid receptor activation, increased both mRNA expression and enzymatic activity of 11β-hydroxysteroid dehydrogenase type 1 (11β-HSD1), being able to accelerate adipocyte differentiation and adipogenesis in human visceral adipocytes [172]. Obesogenic association relied on the rationale that the enzyme 11β-HSD1 converts the inactive hormone cortisone to the active hormone cortisol in adipose tissue, promoting adipogenesis [172]. Indeed, enhanced *PPARG* and *LPL* expression, as well as lipid accumulation, were observed in BPA-treated adipocytes [172]. Gestational exposure to BPA was shown to prominently affect adipogenic gene expression in females during foetal adipogenic differentiation, specifically increasing *pparg*, *cebpa* and *glut4* mRNA levels [173]. In mature adipocytes, BPA upregulated the expression of the lipid metabolism-related genes *FABP4* and *CD36* and the expression of the C–C motif chemokine ligand 20 (*CCL20*), *IL18*, and *IL1B* [174]. BPA-treated adipocytes also presented higher release levels of proinflammatory cytokine [174]. These findings underscore the role of BPA in inducing inflammation of the adipose tissue, which is a hallmark of obesity-related pathophysiology. Accordingly, the proinflammatory potential of BPA has been pointed out as a mechanism of obesogenic disruption, activating proinflammatory signalling in fat cells by upregulating the expression and release of specific cytokines, such as CCL20, IL-18, IL-6, IL-1β, and TNF-α [174, 175]. These alterations, associated with a chronic low-grade inflammatory state, drive the perpetuation of the physiological complications of obesity.

Other reported actions for BPA that exacerbate its disrupting potential as an obesogen encompass the promotion of the self-renewal of adipose tissue macrophages, which was linked to extracellular signal-regulated kinase (ERK) phosphorylation [176]. In HFD-fed animals, BPA exposure accelerated the increase in body weight gain, concurrent with inflammatory responses in the adipose tissue that include an increase in IL-17A and macrophage polarisation towards M1 stage (proinflammatory phenotype) [177].

The BPA substitute BPS, due to its structural similarities with BPA, has also been shown to interfere with adipocyte function and lipid metabolism Table 1, Fig. 2. BPS treatment (0.1–25 µM) induced adipogenesis in murine and primary human subcutaneous and visceral preadipocytes, mimicking the actions of glucocorticoids or PPAR-γ agonists [178–180]. Moreover, BPS (25 µM) modified the adipokine profile of visceral adipocytes, increasing *IL6*, *IL8* and *IL1B* mRNA levels [178]. Curiously, BPS appears to be a more potent inducer of adipogenesis and lipid metabolism (*e.g.* cholesterol biosynthesis pathways) than BPA in both rodent and human subcutaneous preadipocytes, with earlier transcriptional effects and stronger upregulation of involved genes [179–181]. The perinatal and chronic exposure to BPS (1.5–50 µg/kg) impacted lipid homeostasis, inducing overweight in the offspring of HFD-fed male mice, which positively correlated to fat mass, hyperinsulinemia, and hyperleptinemia [182].

Phthalates represent the most common group of plasticisers used globally (~ 75%), primarily in the production of polyvinyl chloride plastics [183]. These broad applications mean that the human exposure associated with the usage of plastic products can occur even in hospital care because phthalates can migrate from plastic medical equipment, such as blood bags, catheters, nasogastric, and intravenous tubes [184, 185]. Apart from being used in the production of plastics, phthalates are an integral part of the composition of many other products, such as cosmetics, paper coatings and paints [184, 185].

Chemically, the group of phthalates includes a class of organic compounds known as phthalic acid esters [183]. Di(2-ethylhexyl) phthalate (DEHP), the most widely used polyvinyl chloride plasticiser, has received considerable attention regarding endocrine disruption and potential obesogenic effects [186–189]. DEHP easily leaches out from plastics during production processes, use, and disposal, as it is combined with the parent materials by a non-covalent bond [190]. Thus, exposure of the general population to DEHP is inevitable, with the dietary route accounting for more than 80% of daily intake in adults [186]. Also, due to the high lipophilicity of this chemical, fatty food is known to be readily contaminated by DEHP [186]. Once absorbed into the intestine and parenchyma, DEHP is metabolised by esterase and lipase into active mono(2-ethylhexyl) phthalate (MEHP), which is considered more toxic due to its potential to activate several nuclear receptors and initiate the downstream cascade effects [191]. Cross-sectional studies have reported a positive correlation between urinary DEHP metabolites and obesity parameters, such as body mass index (BMI) and waist circumference, in children, adolescents, and adults [192, 193]. Indeed, a systematic review with meta-analysis of 31 studies demonstrated that early life exposure to DEHP is strongly associated with increased adiposity in rodents [194]. In vivo studies corroborated DEHP-induced weight gain and fat mass expansion, concomitant with lower adiponectin levels [195–199]. Under HFD conditions, DEHP upregulated the PPAR-γ expression and its phosphorylation at Ser273 in WAT, indicating a synergistic effect between dietary and chemical stressors [198]. MEHP has been demonstrated to directly activate PPAR-γ in 3T3-L1 murine preadipocyte cells and human adipocytes, prompting adipogenesis and gluconeogenesis with increased proliferation [200–202]. MEHP can perturb key regulators of adipogenesis and lipogenic pathways in vivo, increasing *pparg*, adipocyte-specific fatty acid binding protein (*ap2*) and *LPL* expression in peri-epididymal adipose tissue, with elevated levels of serum cholesterol, TGs and glucose [202]. Enhanced proliferation rate and glucose uptake, as well as induced PPAR-γ and C/EBPα expression, were also observed upon DEHP exposure in 3T3-L1 adipocytes [195, 199]. DEHP exposure led to a proinflammatory state in adipocytes, reflected by increased secretion of IL-8 and MCP-1 [203].

### Additives in Food, Cosmetics, and Pharmaceuticals

Integrating the existing evidence, it becomes clear that the primary route of human exposure to obesogens is through the ingestion of contaminated food [14]. The threat rising from this situation is magnified by the global rise in consumption of ultra-processed foods containing numerous chemical additives [14, 204], which have been shown to display obesogenic features [205–211]. The panoply of food-related compounds with potential obesogenic effects includes dietary emulsifiers (carboxymethylcellulose, polysorbate-80, P-80), surfactants (dioctyl sodium sulfosuccinate, DOSS, Span-80), preservatives (3-tert-butyl-4-hydroxyanisole, 3-BHA), and flavour enhancers (monosodium glutamate, MSG) Table 1, Fig. 2 [205–211].

Low concentrations of the dietary emulsifiers carboxymethylcellulose and P-80 have been shown to induce intestinal low-grade inflammation and disrupt the gut microbiome, resulting in increased body weight, WAT depot weight, and metabolic syndrome, regardless of the age of the exposed animals [205, 206]. In the case of the surfactant DOSS, male offspring of pregnant-exposed mice exhibited increased body and visceral fat mass, decreased plasma adiponectin and increased leptin and *il6* [208]. DOSS has also been shown to bind the PPAR-γ ligand-binding domain, acting as a PPAR-γ agonist in reporter assays and inducing adipogenesis in 3T3-L1 preadipocytes [207]. Similarly, the surfactant Span-80 can activate RXRα, inducing 3T3-L1 preadipocytes to differentiate into adipocytes [209]. Curiously, when 3T3-L1 cells were treated simultaneously with Span-80 and DOSS, the adipogenic induction was greater than in individual exposure [209]. Moreover, elevated expression of *cox2* and *nox4* was observed in the WAT of adult F1 male mice in association with the presence of reactive oxygen species and systemic chronic inflammation [208].

In addition to emulsifiers and surfactants, the widely used food preservative 3-BHA also induced 3T3-L1 preadipocyte differentiation concomitantly with enhanced transcription of *pparg*, *cebpa* and *fabp4*[212]. Another study exploiting the effect of 3-BHA on the differentiation of MSCs into brown adipocytes demonstrated that this chemical was responsible for shifting MSC differentiation from a brown to white-like phenotype [213]. Exposure to 3-BHA compromised the thermogenic capacity of the differentiated cells and promoted lipogenesis, evidenced by the increased intracellular lipid accumulation and elevated expression of *pparg*, *perilipin*, *adiponectin*, and *fabp4* [213]. Additionally, exposure to 3-BHA activated Smad1/5/8 phosphorylation in a time-dependent manner, suggesting the involvement of Smad signalling pathways in the mediation of 3-BHA’s effects [213]. Interestingly, the obesogenic effects of 3-BHA were more pronounced in HFD-fed animals, as translated by augmented body and peri-gonadal WAT weight, adipocyte enlargement and increased TG plasma levels compared to HFD-fed mice only [210]. Moreover, the analysis of lipid metabolism and inflammation regulators revealed higher gene expression of the fatty acid transporter *cd36*, *pparg*, *acc*, *hormone-sensitive lipase*, *tnfa* and *il6* in the peri-gonadal WAT of HFD-fed animals exposed to 3-BHA [210].

The flavour enhancer MSG has long been associated with obesity and metabolic abnormalities. Initially, it was believed that MSG exerted obesogenic effects, with increased body and fat weight, TG and cholesterol, exclusively by damaging the hypothalamic arcuate nucleus, which is involved in the regulation of body mass and energy metabolism [211, 214, 215]. More recently, it was shown that MSG might act by other mechanisms, namely by impairing the secretion of glucagon-like peptide-1, an important hormone in regulating appetite [8]. Thus, MSG may play a role in the pathogenesis of obesity by impacting satiety responses and glucose-stimulated insulin release, contributing to weight gain through both central and peripheral mechanisms.

Parabens, used as preservatives in food, cosmetics and pharmaceuticals, are known for their antimicrobial and antifungal properties [216]. These compounds were shown to promote adipogenesis in 3T3-L1 preadipocytes and MSCs by activating PPAR-γ, modulating lipid accumulation and enhancing the mRNA expression of adipocyte-specific markers, such as *pparg*, *cebpa*, *fasn*, *fabp4*, *perilipin* and *adiponectin* [217–219]. Interestingly, the adipogenic potency of parabens is associated with their chemical structure, *i.e.* the length of the linear alkyl chain (methyl- < ethyl- < propyl- < butyl-paraben) and the existence of an aromatic ring in benzylparaben that further augments the adipogenic ability [218]. In MSCs, both methyl and butylparaben were shown to promote adipogenesis while suppressing osteogenic and chondrogenic differentiation, with methylparaben showing less pronounced effects [219]. Butylparaben activated PPAR-γ, whereas its adipogenic effects were significantly attenuated by PPAR-γ knockdown, which strongly implicates the PPAR-γ signalling pathway driving its effects [219]. Despite earlier cross-sectional and longitudinal human studies not finding a consistent association between exposure to parabens and overweight or obesity [220–223], a more recent longitudinal study involving 496 mother–child pairs reported that prenatal exposure to butylparaben was associated with child overweight during the first 8 years of life, with a stronger trend among females [224]. Furthermore, mechanistic analysis revealed that early-life exposure to butylparaben induced higher food intake in the female offspring, which was accompanied by the modulation of appetite regulators, namely the downregulation of leptin receptor and the reduced hypothalamic expression of proopiomelanocortin [224].

Nonylphenol (NP), a degradation product of industrial surfactants, is another additive with obesogenic potential. Although not widely used as a direct commercial ingredient, NP is commonly present in products containing nonylphenol ethoxylates, namely plastics, pesticides, and cosmetics [225]. Perinatal exposure of rodents to NP induced adipocyte differentiation, increased *pparg* expression, promoted the augmentation of body weight, accumulation of fat mass, and the increase of fasting serum glucose and total cholesterol levels in the offspring [226, 227]. NP was shown to stimulate adrenal function and increase 11β-HSD1 activity in liver and adipose tissue, which supports the obese phenotype [227]. Another study reported the NP effect inducing accumulation of TG and the expression of adipogenic markers in MSCs-derived adipocytes [228].

Notwithstanding the reported obesogenic actions, the molecular mechanisms underlying the effect of NP remain far from being fully understood.

### Flame Retardants

Flame retardants are widely used in the manufacturing of everyday products such as electronics, furniture, toys, and foodstuffs [229–231]. Among the diversity of products with flame-retardant properties, polybrominated diphenyl ethers (PBDEs) were the most commonly used until their production and use were phased out in the EU and the USA in 2004 due to concerns about neurotoxicity and metabolic deregulation [232–234]. Nevertheless, PBDEs remain ubiquitous in the environment and consumer goods, with ongoing human exposure, which occurs through ingestion, inhalation, and dermal contact [235–239]. PBDEs are constituted by two benzene rings connected by an ether bond, with varying numbers and positions of bromine atoms attached to the rings, which result in 209 possible PBDE congeners [240]. These compounds are highly lipophilic, and for this reason, accumulate preferentially in the adipose tissue [240]. Accordingly, studies reported that PBDEs levels in adipose tissue positively correlated with visceral fat and visceral/subcutaneous abdominal fat ratio in obese individuals [241, 242]. PBDEs’ levels were determined in adipose tissue samples obtained by liposuction in 98 Czech subjects, with PBDE congeners 47, 99, 153 and 183 being the most abundant, constituting up to 90% of these pollutants in adipose tissue [243]. The bioaccumulation of PBDEs within adipocytes impaired lipid and glucose metabolism, increasing lipolysis and decreasing glucose oxidation, which was associated with enhanced risk of metabolic disease, including obesity [244–246]. An *in vivo* study demonstrated that perinatal exposure to decabromodiphenyl ethane (DBDPE) increased obesity risk in male mice offspring, affecting lipid and glucose metabolism, effects exacerbated in HFD conditions [247]. Also in humans, a positive correlation between serum levels of the flame retardant PBDE153, visceral fat mass and metabolic syndrome was established [248]. The levels of 28 PBDE congeners were also assessed in subcutaneous and visceral adipose tissues from 34 obese individuals to determine the correlation with metabolic disease [249]. Out of the detectable PBDEs, PBDE28, 47, 99 and 153 were predominant in visceral adipose tissue, and PBDEs 28, 47 and 99 were significantly higher in insulin-resistant individuals compared to insulin-sensitive counterparts [249]. Treatment of human visceral preadipocytes from insulin-sensitive individuals with PBDE28 inhibited the phosphorylation of glycogen synthase kinase 3 (GSK3) α/β, mammalian target of rapamycin (mTOR), p70 S6 kinase and S6 ribosomal protein, and PTEN activation was observed [249]. These findings suggest the inhibition of insulin signalling and a relationship between PBDEs accumulation in human adipose tissue and insulin resistance in obese individuals [249]. Concerning the molecular targets reached, a mixture of PBDEs and BDE-47 enhanced the expression of adipogenic markers (*fabp4*, *perilipin*, *cebpa*, *pparg*, *lxra*) and increased TG storage in 3T3-L1 preadipocytes, even in the absence of glucocorticoids (dexamethasone) [250]. Moreover, epigenetic studies have revealed that BDE-47 induced demethylation of CpG sites in the *pparg* promoter, further supporting its role in adipogenesis by increasing the gene expression of *pparg*, *leptin*, and glucose-6-phosphatase catalytic subunit *(g6pc)* in differentiated 3T3-L1 adipocytes [251].

Recently, organophosphate flame retardants (OPFRs) emerged as a new class of compounds aiming for the replacement of PBDEs [231]. Despite the already existing data on the toxicity of these flame retardants at high concentrations, OPFRs became widely used among home furnishing manufacturers, resulting in widespread human exposure [229, 252]. OPFRs are embedded in household and workplace products, being easily released from materials (*e.g*. by abrasion, leaching, or volatilisation) into dust, as they are only physically added to materials and not chemically bonded to them [229, 252]. Thus, OPFRs exposure is more prevalent in indoor air than in the outdoors [253]. Human exposure occurs primarily via inhalation and ingestion, resulting in biologically relevant levels (1–10 ng/mL) in human serum and urine samples [252, 254–257]. OPFRs are not yet reported to accumulate in the adipose tissue to the same extent as PBDEs. However, the hydrophobicity of aryl OPFRs makes this a highly likely possibility [231, 258, 259]. In a recent study including 188 obese women, five out of seven investigated organophosphate esters (OPEs) were detected in both subcutaneous and visceral fat, with a slightly higher frequency in visceral adipose tissue (53.2% of the analysed samples) [260]. Furthermore, the ability of OPFRs to interact with nuclear receptors involved in hormonal signalling and in the pathogenesis of metabolic syndrome (*e.g.* PPAR-γ, ERα, AR, farnesoid X receptor, FXR, pregnane X receptor, PXR) [261–265] was demonstrated, which raises concern over potential long-term adverse health effects. Epidemiologic studies evaluating the obesogenic potential of OPFRs in humans are still emerging. Bis(1-chloro-2-propyl) phosphate (BCPP) was positively associated with obesity in adults, along with metabolic dysregulation and altered total cholesterol levels [260, 266, 267]. In a study comprising 1334 adults, the association between urinary metabolites of OPFRs and BMI was investigated [266]. It was demonstrated that a one-unit increase in log_2_-transformed urinary concentration of bis(2-chloroethyl) phosphate (BCEP) and bis(1,3-dichloro-2-propyl) phosphate (BDCPP) was associated with 0.27 and 0.56 higher BMI value, and 1.1- and 1.19-fold risk for developing obesity, respectively [266].

Recent studies conducted in distinct biological models (*e.g.* cells, zebrafish, and mice) have disclosed the alterations occurring in the obesity-like dysregulation induced by exposure to OPFRs, and revealed the links between OPFRs exposure and disruption of lipid metabolism, OS, and inflammatory responses [265, 268–270]. OPFR treatment has revealed sex- and/or diet-dependent alterations in physical activity, ingestive behaviour, metabolism and fat accumulation [265, 271]. In an experimental model using adult mice exposed to a mixture of OPFRs (triphenyl phosphate, TPP, tricresyl phosphate, TCP, and tris(1–3-dichloro-2propyl)phosphate, TDCPP, 1 mg/kg of each), on a low-fat diet (LFD) or HFD, males exhibited elevated activity and oxygen consumption, while in females these parameters were decreased, irrespective of diet [271]. OPFRs increased body weight gain only in HFD males, increasing circulating insulin (LFD) and leptin (HFD) in females and decreasing ghrelin in males (LFD) [271]. Tenlep et al*.* also demonstrated that TDCPP caused male-specific adiposity, insulin resistance and fasting hyperglycaemia [265]. In zebrafish, the excess lipid accumulation verified after tris(1-chloro-2-propyl)phosphate (TCPP) treatment was concomitant with enhanced adipogenesis and suppressed fatty acid β-oxidation, triggered OS, and induced the overexpression of proinflammatory cytokines (*il1b*, *il22*) [269]. Also, tris (2-chloroethyl) phosphate (TCEP) induced body weight gain and hyperlipidaemia in adult mice, consistent with the upregulation of hepatic lipogenesis-related gene expression [270]. In silico, *in vitro*, and *in vivo* assays assessing 2-ethylhexyl diphenyl phosphate (EHDPP), triphenyl phosphate (TPHP), and TCP exposure suggested that aryl-OPFRs act as noncompetitive inhibitors of adiponectin receptors, impairing their metabolic signalling [272]. In alpha mouse liver 12 cells exposed to aryl-OPFRs, this adiponectin signalling dysregulation culminated in metabolic disturbance with lower glucose uptake and higher lipid content [272]. In mice, exposure to TCP suppressed adiponectin receptor 1 signalling, which was marked by lower levels of phosphorylated AMPKα and higher expression of gluconeogenesis-related genes [272]. TPHP is another OPFR with demonstrated obesogenic potential, as indicated by the increased body and liver weight, accumulation of fat mass, impaired glucose homeostasis and insulin resistance upon exposure of mice to this compound (1 mg/kg) [273]. The reported physiological alterations were underpinned by the increased mRNA levels of lipid metabolism-related genes (*pparg*, *fasn*), favouring lipogenesis and lipid accumulation [273].

Globally, the summarising data highlight the increasing risk posed by flame retardants, both legacy PBDEs and their OPFR replacements, as emerging contributors to metabolic dysregulation and obesity Table 1, Fig. 2. Their ability to disrupt endocrine and metabolic pathways substantiates their classification as environmental obesogens.

## Effect of Obesogens Targeting Prostate Cells

The hormone-dependent nature of PCa, combined with the growing recognition of environmental influences on its pathophysiology, highlights the potential role of EDCs in its initiation and progression [21, 274–280]. As has been discussed, besides triggering endocrine impairment, EDCs with obesogenic potential can also disrupt metabolic processes. Moreover, endocrine and metabolic regulation are closely interconnected in the control of several biological processes, and metabolic reprogramming is one recognised hallmark of cancer [26, 102, 281]. For this reason, obesogens represent a dual threat to PCa, with effects possibly exacerbated relative to other EDCs, driving alterations in prostate cells through both endocrine and metabolic alterations.

### Cell Survival and Invasiveness

**Fig. 3:**
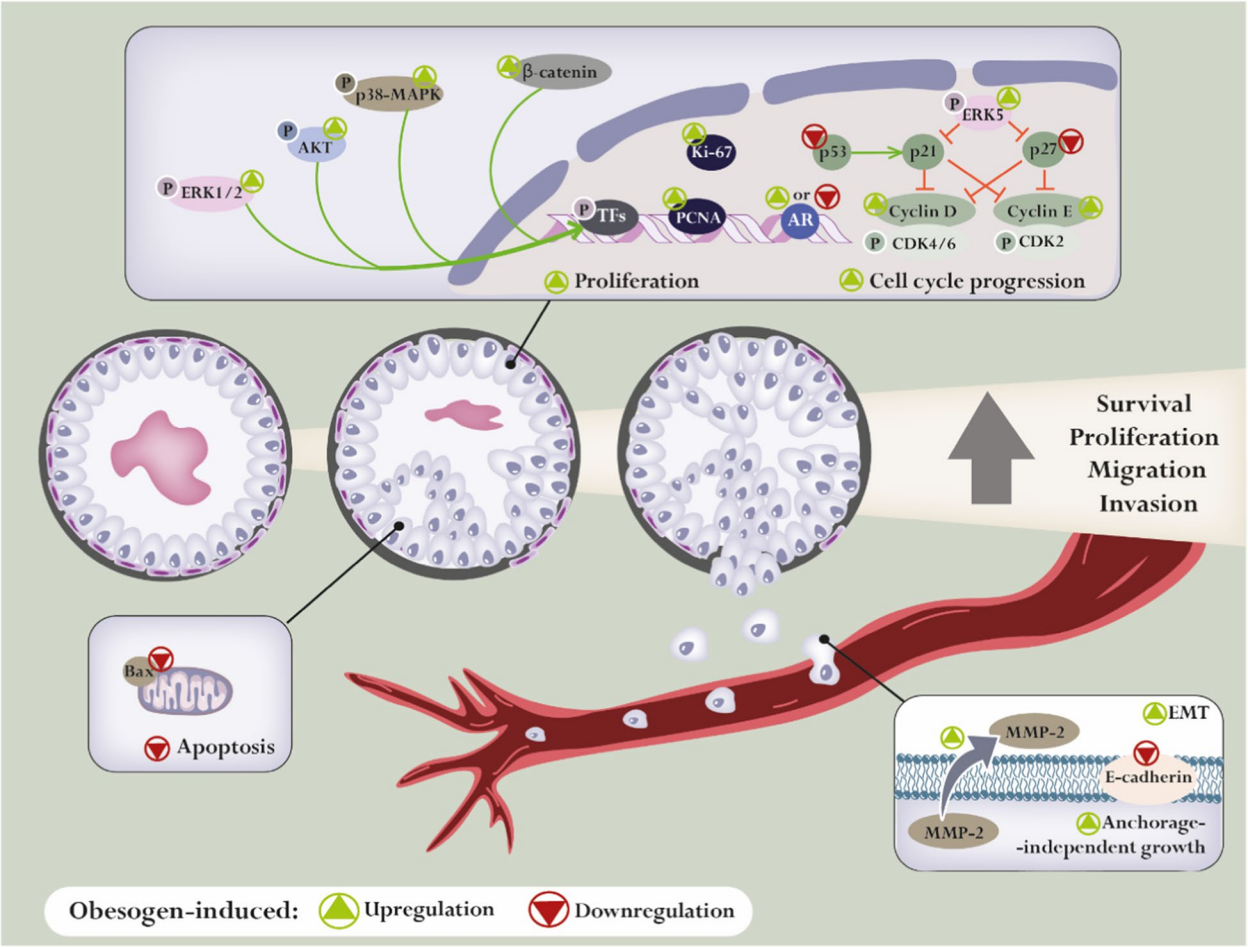
Effect of obesogens targeting prostate cell fate. Studies exploring the mechanisms of obesogen-induced dysregulation in prostate cells have focused on hormonal signalling (*e.g.* up/downregulation of the androgen receptor, AR, function), as well as on alterations in proliferation, cell cycle, apoptosis and epithelial-to-mesenchymal transition (EMT) pathways. Obesogens can promote pro-carcinogenic features in the prostate, including increased survival, proliferation, migration and invasion of prostate cancer cells. AKT: protein kinase B; CDK: cyclin-dependent kinase; ERK: extracellular signal-regulated kinase; MAPK: mitogen-activated protein kinase; MMP: matrix metalloproteinase; PCNA: proliferating cell nuclear antigen; TFs: transcription factors

Studies demonstrating the capability of obesogens to disrupt prostate cell fate are scarce and mainly focused on hormone signalling. Table 2 and Fig. 3 summarise the relevant existing information. The effects of organotins (TBT, TPT and DBT) have been studied. Both TBT (100 nM) and TPT (1 nM) enhanced AR-dependent transcription, expression of the AR-target gene prostate-specific antigen (*PSA*), and cell proliferation of LNCaP-derived LA16 PCa cells, which stably expressed an androgen-responsive luciferase reporter gene [15]. Organotins’ effects in promoting LA16 cell proliferation were to the same extent as those of the potent androgen, dihydrotestosterone (DHT, 1 nM) [15], which corroborates that the activation of AR mediates their actions. Curiously, TBT, TPT and also DBT can also dysregulate DHT biosynthesis, as it was demonstrated that they have the capability to inhibit 5α-reductase activity in LNCaP cells, with IC_50_ of 2.7, 4.2 and 11.2 µM, respectively [16].

**Table 2:** Obesogens with reported direct effects on prostate cell fate

Obesogen	Cell model	Effect	Refs
TBT	LA16 cell line	↑ AR-dependent transcription ↑ PSA expression	[94]
	LNCaP	⊸ 5α-reductase activity	[95]
DBT (TBT metabolite)	LNCaP	⊸ 5α-reductase activity	[95]
TPT	LA16 cell line	↑ AR-dependent transcription	[94]
	LNCaP	⊸ 5α-reductase activity	[95]
DDT	LNCaP	↓ AR-dependent transcription ↓ PSA, PMSA, IGF-1 expression	[96]
	VCaP	↓ AR-dependent transcription ↓ PSA, PMSA, IGF-1 expression	[96]
DDE (DDT metabolite)	LNCaP	↑ Proliferation ↓ AR-dependent transcription ↓ PSA, PMSA, IGF-1 expression ↑ Activity of protein tyrosine kinases ↑ Expression of MAPK	[96, 97]
	VCaP	↓ AR-dependent transcription ↓ PSA, PMSA, IGF-1 expression	[96]
NP	PNT1A	↑ Proliferation ↑ Expression of cyclin D, cyclin E and ki-67 and the pro-inflammatory cytokine IL-1β	[98]
	LNCaP	↑ Viability, proliferation and migration ↑ Expression of cyclin D, cyclin E and ki-67 and the pro-inflammatory cytokines IL-1β and IL-8 ↓ Expression of p27 and Bax	[99, 100]
	DU145	↑ Proliferation ↑ Expression of GPER	[101]
Dichlorvos	LNCaP	↑ Viability ↑ Nuclear AR translocation and expression	[102]
DEHP	22Rv1	↑ Viability ↑ Cyclin D1, PCNA, ERK5, p-p38 MAPK and activator protein 1	[103]
	DU145	↑ Proliferation and migration ↓ Levels of p53 and increased levels of β-catenin	[104]
	PC3	↑ viability ↑ Expression of cyclin D1, PCNA, ERK5, p-p38 MAPK and activator protein 1	[103]
MEHP (DEHP metabolite)	LNCaP	↓ Proportion of DNA cytosines with 5-methylcytosine methylation	[105]
BPA	C4-2	↑ Anchorage-independent growth	[106]
	22Rv1	↑ proliferation and invasion capacity ↑ Transcriptional activity of AR mutants (AR-T877A, AR-T877S, AR-V715M and AR-H874Y)	[107, 108]
	LNCaP	↑ Proliferation, migration and invasion capacity	[107–109]
	LAPC4	↑ Proliferation and invasion capacity	[107, 108]
	Human prostate stem cells	↑ Self-renewal and maintenance of a stem-like phenotype ↑ Phosphorylation of AKT and ERK	[110]
BPS (BPA substitute)	C4-2	↑ Proliferation	[111]
	LNCaP	↑ Proliferation	[111]
Chlorpyrifos	DU145	↑ EMT Reduced E-cadherin expression ↑ Migration, anchorage-independent survival and MMP-2 release ↑ Levels of phospholipids, glycosphingolipids, TG, and cardiolipins	[112]
OPFRs (TPP)	LNCaP	↑ Proliferation and invasion capacity	[113]
	PC3	↑ Proliferation and invasion capacity	[113]

*↑* Increased/stimulated, *↓* decreased, ⊸ Inhibited, *AKT* Protein kinase B, *AR* Androgen receptor, *BPA* Bisphenol A, *BPS* Bisphenol S, *DBT* dibutyltin, *DDT* dichlorodiphenyltrichloroethane, *DEHP* Di(2-ethylhexyl) phthalate, *EMT* Epithelial-to-mesenchymal transition, *ERK* Extracellular signal-regulated kinase, *GPER* G protein-coupled oestrogen receptor, *IGF-1* Insulin-like Growth Factor-1, *IL* Interleukin, *MAPK* Phosphorylated mitogen-activated protein kinase, *MEHP* Mono(2-ethylhexyl) phthalate, *MMP* Matrix metalloproteinase, *NP* Nonylphenol, *OPFRs* Organophosphate flame retardants, *PCNA* Proliferating cell nuclear antigen, *PMSA* Prostate-specific membrane antigen, *PSA* Prostate-specific antigen, *TBT* Tributyltin, *TG* Triglyceride, *TPP* Triphenyl phosphate, *TPT* Triphenyltin.

Androgens, through AR-mediated signalling, play a crucial role in the growth, function, and survival of prostate cells, stimulating proliferation and inhibiting apoptosis [280, 282]. However, the pesticide DDT and its main metabolite DDE (both at 10 µM) negatively impacted AR-regulated expression of *PSA* and other AR target genes (prostate-specific membrane antigen, *PMSA*, and Insulin-like Growth Factor-1, *IGF1*) in human LNCaP and VCaP PCa cells, blocking the recruitment of AR to the PSA promoter region [283]. These antiandrogenic actions of DDT and DDE are little known and apparently would suppress PCa growth. However, the authors of the study launched the idea that prolonged exposure to these EDCs may be as noxious as androgenic exposures, as it mimics the scenario of androgen deprivation therapy (ADT), exacerbating cancer development by potentially promoting the early emergence of castration resistant PCa [283]. This is corroborated by the fact that DDT was shown to increase the activity of protein tyrosine kinases in the androgen-dependent LNCaP PCa cells, enhancing their proliferative activity and the expression of MAPK [17].

The fact that an EDC is mainly recognised by its ability to disrupt a specific hormone signalling does not necessarily mean that its action is limited to the deregulation of that pathway alone. The well-recognised xenoestrogen NP, which was demonstrated to activate both ER- and AR-dependent signalling pathways in prostate cells [284–286], is an example. Increased proliferation was observed in the prostate cell lines PNT1A (non-neoplastic) and LNCaP (adenocarcinoma) after exposure to NP (10 µM and 1 nM, respectively), with enhanced cytoplasm-nucleus translocation of ERα (and not ERβ) and increased gene expression of key cell cycle regulators (cyclin D (*CCND1*), cyclin E *(CCNE1)* and *ki67*) and proinflammatory cytokines (*IL1B* in both, *IL8* in LNCaP) [284, 285]. The upregulation of proinflammatory cytokines in more than 50% indicated a major involvement of NP in dysregulating inflammatory processes [284, 285]. In another study, NP (0.01–1 µM) promoted the proliferation of androgen-insensitive PCa cells (DU145), with concomitant increased expression of G protein-coupled oestrogen receptor [287]. Notably, Forte M et al. [285] demonstrated that the ER antagonist ICI182780 only partially reverted the observed effects of NP, confirming the estrogenic activity of NP but also supporting a dual AR/ER mode of action in the prostate [285]. Interestingly, NP (0.1–100 µM) increased LNCaP viability and migration similarly to DHT (10 nM), an effect reversed by the AR antagonist Casodex, suggesting that NP can indeed act via the AR signalling pathway [286]. To elucidate the mechanisms underlying NP actions in LNCaP, this study evaluated the transcriptional levels of cell cycle- and apoptosis-related markers, demonstrating increased expression of cyclins D1 and E and reduced mRNA levels of p27 (*CDNK1B*) and *BAX* [286].

The OP dichlorvos, considered an estrogenic EDC, also mimics androgen function in LNCaP cells, increasing AR expression and its translocation to the nucleus [288]. Moreover, it modulated the expression of epigenetic regulators, namely DNA methyltransferase 1 (DNMT1) and histone deacetylase 1 (HDAC1), enhancing cell viability across all tested concentrations (10, 50 and 100 nM) [288].

Actions reported to affect prostate cell fate in the interplay with epigenetic alterations were also reported for other classes of EDCs. The DEHP’s active metabolite MEHP seems to affect the progression of PCa through its effect on global DNA methylation, decreasing the proportion of cytosines with 5-methylcytosine methylation in LNCaP cells, in a concentration-dependent manner (1–25 μM) [289]. DEHP (100 nM) was shown to increase the viability of 22Rv1 and PC3 cells, upregulating the proliferation inducers cyclin D1 and proliferating cell nuclear antigen (PCNA) [290]. Furthermore, upregulated expression of p-ERK5 and p-p38 MAPK, along with activator protein 1 (AP-1: p–c-fos and p–c-jun), was observed [290]. Using ERK5 and p38 inhibitors, data showed that the downregulation of p-ERK5 or p38 inhibited phthalate-triggered cell proliferation, suggesting that the activation of the MAPK/AP-1 pathway by phthalates may potentially promote PCa cell proliferation [290].

In turn, the plasticiser BPA was shown to affect epigenetic regulators such as histone deacetylase SIRT1, and the histone methyltransferase SET8 in PCa cells [291]. BPA is another example of a xenoestrogen with pleiotropic effects dependent on AR status, as it promotes the activation of AR-T877A, leading to androgen-independent PCa cell proliferation [292]. These findings support the idea that BPA may facilitate the progression of prostate tumours to the ADT-resistant stages, influencing the duration and magnitude of therapeutic response in PCa patients. BPA actions on different tumour-derived AR mutants were concentration-dependent and displayed distinct effects in different PCa cells [292]. At low concentration (10 nM), BPA stimulates the transcriptional activity of AR-T877A, and this effect is amplified in the presence of physiological androgen concentrations (10 nM) [292]. BPA is also able to activate or potentiate the transcriptional activity of other functional AR mutants such as AR-T877S, AR-V715M and AR-H874Y (isolated from human prostate carcinoma xenograft-derived 22Rv1 cells), but does not affect wild-type AR [292]. Curiously, BPA (1 µM) was shown to increase the proliferation and invasion capacity of PCa cells, regardless of expressing mutated (LNCaP, 22Rv1) or wild-type AR (LAPC4) [292, 293]. This can be easily justified as another interesting peculiarity of BPA action is that, besides activating transcription pathways similarly to DHT, it also induces ERβ down-regulation (not observed following DHT stimulation), followed by induced PCa cell proliferation even under androgen-deprived conditions [294]. Studies on human stem/progenitor cells derived from prostates of young, disease-free human donors showed that BPA increases self-renewal and maintenance of their stem-like nature in a dose-dependent manner, and triggers the rapid phosphorylation of AKT and ERK [295]. Also, the BPA substitute BPS (1 µM) was shown to increase the proliferation of C4-2 and LNCaP PCa cells [296], showing that the new plasticisers can be as noxious as BPA in prostate carcinogenesis.

Tumour cell migration and invasion, driven by processes such as epithelial-to‑mesenchymal transition (EMT), invadopodia formation, and extracellular matrix remodelling, are pivotal mechanisms enabling tumour progression and metastasis [26]. In DU145, DEHP (20–200 nM), besides increasing cell proliferation, also enhanced migration and increased β-catenin levels, while reducing p53 expression [297]. Indeed, obesogenic compounds have also been shown to stimulate the migratory capacity and invasiveness of PCa cells. In this context, chlorpyrifos was investigated as an enhancer of DU145 aggressiveness after chronic exposure (50 days, 1 µM) [298]. Exposure to chlorpyrifos stimulated EMT as translated by a 14% loss of E-cadherin expression, increased migration (26% higher), anchorage-independent survival (44% higher) and matrix metalloproteinase 2 (MMP-2) release (37% higher) compared to the unexposed cells [298]. BPA exposure also seems to be involved in this process, strongly stimulating cell migration (LNCaP cells, 1 or 10 nM) and anchorage-independent growth (C4-2 cells, 0.1 nM) [299, 300]. The OPFR TPP (10 µM) significantly increased both cell proliferation and invasion capacities in LNCaP and PC3 cells, concomitantly with upregulation of *AR*, *MTOR* and DNA damage-inducible transcript 3 (*DDIT3*) [301].

Although obesogens can modulate prostate cell proliferation, migration, and invasion, evidence of effects on non-neoplastic cells is limited. Most studies rely on metastatic PCa models, which makes it difficult to definitely conclude about the carcinogenic role of obesogens in healthy prostate tissue. Studies are warranted to address this scientific gap and clarify the potential contribution of obesogens to PCa initiation.

### Metabolic Alterations

Most studies focus on hormonal disruption induced by EDCs/obesogens, with few addressing their actions in shaping the metabolic reprogramming of prostate cells towards carcinogenesis. In this context, lipidomic analysis in DU145 cells exposed to chlorpyrifos revealed increased levels of phospholipids, glycosphingolipids, TGs, and cardiolipins, suggesting a link between chlorpyrifos-deregulated lipid metabolism and malignancy [298].

Altered lipid metabolism characterised by the reprogramming of de novo fatty acid biosynthesis and augmented b-oxidation, driven by increased expression of FASN and enhanced mitochondrial activity, is a common event in the development and progression of cancer [281]. Quintino-Ottonicar et al. evaluated the influence of the pesticide dichlorvos (10 mg/kg) on the lipid metabolism of rat prostate after chemical carcinogenic induction by N-methyl-N-nitrosourea (MNU) [302]. Enhanced expression of the lysosomal integral membrane protein-II (LIMP II), a molecule correlated with the capture and distribution of lipids in tumours, was observed, concomitantly with 100% incidence of epithelial hyperplasia and increased epithelial compartment in the ventral prostate of dichlorvos-exposed animals [302]. Further research is needed to elucidate the mechanisms by which obesogens alter prostate metabolism, promoting cancer development.

### What Can Be Learned From *In Vivo* Models

*In vivo* animal models are valuable tools for assessing the impact of obesogenic compounds on prostate physiology and malignant transformation. As an advantage, these models enable translating the complexity underlying the action of obesogens in disrupting cell behaviour into a context of real exposure, considering the influence of the tumour microenvironment (TME), and interorgan communication.

Long-term exposure (6 months) of 100-day-old gerbils to BPA at a low dose (50 µg/kg/day) in combination with HFD strongly increased the incidence of premalignant and malignant prostatic lesions with inflammatory infiltration in a rodent model for PCa [303]. Another study showed that neonatal exposure to BPA induced metaplasia, hyperplasia and inflammation, altering prostate morphogenesis and increasing the susceptibility to develop precancerous prostatic lesions, with the development of PCa in adult and elderly life [304–306]. It was also described that gestational exposure to BPA (25 and 250 μg/kg) causes histological alterations in the rat prostate observed at the postnatal day 180 [307]. BPA induced the formation of a multifocal inflammatory infiltrate in the ventral and dorsolateral prostate, with associated papillary prostatic intraepithelial neoplasia (PIN) [307]. It also caused a folded acinar epithelium with reduced acinar lumen and thinner reticular fibres, due to the presence of inflammatory infiltrate, collagen accumulation among the inflammatory cells, and rearrangement of the stroma [307].

Other reports described the effect of phthalates. *In-utero* and lactational exposure to DEHP (0.01–1.01 mg/kg/day) modulated the susceptibility of male offspring to prostate carcinogenesis [308]. Increased PSA concentrations were underpinned by results of PCa grade parameters, including higher PIN and Gleason scores, which meant that exposure to DEHP translated to an increased risk of prostate carcinogenesis [308]. Another study from the same research group concluded that transcriptional changes of glutathione S-transferase (*gstp1*), prostate stem cell antigen (*psca*), and prostaglandin-endoperoxide synthase 2 (*ptgs2*) induced by DEHP exposure might increase the predisposition to prostate carcinogenesis later in life [309].

It is highly plausible that obesogens exert their tumorigenic effects by directly impacting prostate cells but also through the modulation of the TME and the surrounding environment and tissues. Considering the impact of these compounds on adipose tissue, triggering phenotypic changes and altering its secretory activity (Chapter 2), and the relationship of PCa with adiposity, it is reasonable to assume that the procarcinogenic effects of obesogens are primed by the adipose tissue. Moreover, the prostate is an organ surrounded and intimately in contact with adipose tissue, the periprostatic adipose tissue (PPAT), which also supports the hypothesis that obesogen-deregulated PPAT is a driving force in the development of PCa.

## Obesogenic Dysregulation of Adipose Tissue and Prostate Cancer

The molecular mechanisms linking obesity and cancer include metabolic alterations, changes in hormone levels, and the release of inflammatory cytokines by the “obese” adipose tissue [310]. As discussed in the previous sections, obesogenic signalling induces a spectrum of dysfunctional changes in adipose tissue that encompass pathological remodelling of adipose depots via adipocyte hypertrophy, lipid dyshomeostasis, dysregulated secretion of adipokines, namely leptin, and low-grade chronic inflammation [311]. Collectively, these changes foster a systemic and tissue milieu rich in metabolic, hormonal and inflammatory signals that may potentiate tumour proliferation and invasion [311]. In the prostate-specific setting, it is also well-established that “obese” PPAT itself and adipocyte-secreted factors increase the proliferation and invasion of PCa cells, fuelling cancer [312–316]. Although a recent study described tumour-suppressive roles of this adipose depot [317], most of the existing evidence demonstrates the tumour-promoting actions of PPAT [318–322]. This chapter will explore the obesogen-induced changes in adipose tissue, discussing their putative impact on shaping the behaviour of prostate cells towards cancer development.

### Lipid Availability

Adipose tissue serves as the primary reservoir for TGs, and the ability of adipocytes to mobilise and release stored fatty acids via lipolysis, both locally and systemically, is well established and further exacerbated in obesity [29–31]. Similarly, the dysregulation of adipose tissue by obesogens often culminates in lipid dyshomeostasis, characterised by elevated levels of TGs, total and LDL cholesterol, as described in Chapter 2 [138–140, 193, 214, 246, 323]. The capacity of adipose tissue to supply fatty acids to prostate cells has already been demonstrated. Tokuda et al. demonstrated that coculturing adipocytes with PC3 cells increased their proliferative rate, resulting in larger and more frequent lipid droplets within the cytoplasm of the cocultured cells [324]. Another study, using Fourier transform infrared microspectroscopy to analyse isotopically labelled fatty acids, reported their translocation from regional pelvic adipocytes to PCa cells, with higher lipid hydrocarbon signal intensity correlated with their proximity to the adipocytes [325]. This finding is particularly relevant, as obesogenic exposure promotes lipid accumulation in adipose tissue, and PCa cells are known to undergo metabolic rewiring in response to changes in lipid availability [326, 327].

The cellular uptake of free fatty acids, whether derived from TG hydrolysis or adipocytes, requires their transport across the plasma membrane [328]. The precise mechanisms and protein mediators involved in this process remain incompletely understood. However, some molecular players have been identified in mediating cell fatty acid uptake, such as CD36, membrane-associated FABP, and FATPs [328]. Despite some uncertainty regarding the mechanisms by which cells incorporate fatty acids, evidence exists linking fatty acid availability to PCa cell responses. Higher levels of free fatty acids were found in the serum of PCa patients, and *in vitro* experiments have demonstrated that high levels of fatty acids can stimulate the proliferation, migration and invasion of both androgen-sensitive and -insensitive PCa cells (22Rv1 and PC3, respectively), partly by affecting their energy metabolism, which is supported by the observed upregulated expression of PPAR-γ [329]. The increased expression of vimentin and vascular endothelial growth factor (VEGF) corroborates the increased migration and invasion activity [329].

Cholesterol dysregulation has also been implicated in PCa. Cholesterol comprises approximately one-third of plasma membrane lipids, regulating membrane integrity, fluidity, and permeability, and is essential for cell growth, as the duplication of the cell membrane is a prerequisite for cellular proliferation [330, 331]. Additionally, cholesterol is a precursor in the biosynthesis of steroid hormones, including glucocorticoids, mineralocorticoids, and sex hormones and, for this reason, contributes to the intra-tumoral synthesis of androgens [327, 331–334]. PCa cells were shown to have higher concentrations of cholesterol in the nucleus, membrane, and cytoplasm compared to non-neoplastic counterparts [330, 334]. Moreover, the mRNA levels of 3-hydroxy-3-methylglutaryl-coenzyme A (HMG-CoA) reductase (*HMGCR*), an enzyme that catalyses a crucial step in the biosynthesis of cholesterol (conversion of HMG-CoA to mevalonic acid), are higher in PCa and associated with earlier biochemical recurrence of the disease [335]. Studies investigating the expression of transcriptional factors that regulate cholesterol homeostasis further suggest that cholesterol plays a role in prostate carcinogenesis. The expression of the SREBP2 is typically low in non-neoplastic prostate tissue, increasing with tumour aggressiveness in correlation with poor clinical outcomes [336, 337].

Cellular cholesterol homeostasis is maintained within the physiological range by a complex interplay among synthesis, uptake, efflux, and esterification. The majority of exogenous cholesterol is transported in LDL particles, which are internalised upon binding to the LDL receptor (LDLR) [338, 339]. A study reported the accumulation of cholesteryl ester in high-grade and metastatic human PCa but not in normal prostate, prostatitis, BPH and PIN tissues [340]. Notably, this accumulation did not result from de novo synthesis, but rather from enhanced LDL uptake [340]. Recent reports from our research team and others further demonstrated that PCa cell growth is influenced by extracellular lipid levels and LDL availability [327, 341]. Our results showed that LDL-cholesterol supplementation increased the viability, proliferation, and migration of PCa cells, which was enhanced by the presence of DHT [327], highlighting the importance of the endocrine and metabolic connection. Accordingly, LDL-derived cholesterol, internalised via endocytosis and stored in lipid droplets as cholesteryl esters, was shown to be essential for PCa cell growth [341].

Many important regulators of cell growth, cell adhesion, migration, and apoptosis, such as epidermal growth factor receptor (EGFR), MAPK, Src family kinases, protein kinase C, caveolins, and others, are located in membrane lipid rafts [342]. Curiously, PCa cells have more lipid rafts expressing more LDLRs and cholesterol channels on the surface of mitochondria than benign prostate hyperplasia and non-neoplastic prostate cells [330, 334, 342].

The presented evidence supports the hypothesis that enhanced lipid availability in consequence of obesogenic disruption is a trigger mechanism promoting prostate carcinogenesis and/or driving tumour growth. It also brings relevance to addressing this question in future research studies.

### Altered Levels of Leptin, The “Obese” Hormone

Leptin is a pleiotropic hormone primarily produced in WAT, which plays a crucial role in regulating appetite, energy balance, and metabolism [343, 344]. Smaller quantities of leptin are detected in other body tissues apart from WAT, namely brown adipose tissue, placenta, stomach, muscles, bone marrow, and brain [343, 344]. Leptin circulates in the blood in both free (biologically active form) and protein-bound forms, where the equilibrium between free and bound leptin regulates leptin bioavailability [344, 345]. Hyperleptinemia is a common feature of obesity, and serum leptin levels are strongly positively associated with the percentage of body fat [346, 347]. Additionally, as described in Chapter 2 of the present review, it is already known that several obesogenic compounds, such as BPS, DOSS, PBDEs and OPFRs, increase leptin gene expression in adipose tissue and its serum levels [182, 208, 251, 271].

Although the exact molecular mechanisms linking leptin to carcinogenesis are still being explored, increasing evidence suggests that elevated leptin levels may contribute to higher cancer risk. Leptin receptors are found on many types of cancer cells, including breast, ovarian, and prostate cancers, and their expression tends to rise with tumour progression [348–350], significantly increasing the magnitude of leptin cancer-promoting actions.

The mechanisms underlying the effects of leptin in PCa cells encompass distinct signalling pathways. A high leptin concentration (100 ng/mL) promoted migration and EMT in PCa cells DU145 and PC3 via stimulation of the STAT3 pathway [351]. Additionally, a positive association was observed between leptin receptor mRNA expression and poor PCa prognosis [351]. In another study in DU145, leptin was shown to stimulate proliferation, promote invasion and enhance anti-apoptotic signalling through the ERK1/2 signalling pathway [352]. Curiously, in the presence of MAPK and PI3K inhibitors, leptin-induced migration of PCa cells was inhibited by 50% to 70%, highlighting the importance of these pathways in mediating leptin’s oncogenic actions [353]. Moreover, leptin has been shown to upregulate the expression of angiogenic and pro-tumorigenic factors**,** including VEGF, transforming growth factor-β1 (TGF-β1), and basic fibroblast growth factor (bFGF) in both DU145 and PC3 cells [353].

Considering the growing body of evidence demonstrating the ability of leptin to stimulate cancer cell growth, promote a pro-inflammatory TME, and support the development of metastasis, together with the fact that obesogens increase leptin expression and its circulating levels, indicates that this pathway may represent a key indirect mechanism by which obesogenic exposure stimulates the development and aggressiveness of PCa.

### Adipose Tissue Immunomodulation

As previously discussed in Chapter 2, exposure to obesogenic compounds, mainly plasticisers, was demonstrated to dysregulate the secretion of a panoply of cyto/chemokines by adipose tissue, which includes the upregulation of proinflammatory mediators linked to the prostate carcinogenic process, namely several ILs (IL-1β, −6, −8, −17A, −18), TNF-α, MCP-1 and CCL20 [174, 175, 177, 178, 203]. Also, the extensive C–C motif chemokine superfamily and its dysregulation are recognised as key factors in cancer progression [354]. Besides impacting adipose tissue in general, such immunological alterations, if occurring in PPAT, are expected to significantly affect prostate cell fate. For example, CCL7, which is upregulated in “obese” PPAT, has been highly implicated in the aggressiveness of PCa, through the activation of the C–C chemokine receptor type 3 (CCR3) [314]. Very recently, our research group demonstrated that exposure to TBT disrupts the CCL7-CCR3 axis with TBT-treated PPAT secreting high levels of CCL7, which enhanced survival and migration of CCR3-expressing non-neoplastic and neoplastic prostate cells [355]. On the other hand, BPA, in particular, has been shown to promote the self-renewal of adipose tissue macrophages and their polarisation towards the M1 stage [176, 177]. Generally, two major mechanisms can be envisioned to claim obesogens as prostate carcinogens: i) paracrine signalling by proinflammatory cyto- and chemokines produced by the adipose tissue/PPAT under obesogenic stimulation, creating a chronic inflammatory microenvironment that promotes cancer cell survival, facilitates immune evasion, and supports the spread of malignant cells to distant organs; ii) macrophages in the TME (tumour-associated macrophages, TAMs) can adopt different activation states that either promote inflammatory responses (M1) or immune suppression (M2), both paradoxically able to enhance tumour growth depending on the physiological context [356–358].

Among the cytokines identified as being dysregulated by obesogens, IL-6 has been the most extensively studied in the context of PCa [359]. The first indications showed that IL-6 serum levels are elevated in patients with untreated metastatic or castration-resistant PCa compared to those with localised disease or healthy patients [360–362]. Moreover, elevated IL-6 levels have been associated with bone metastases and shorter survival times [363, 364]. Binding of IL-6 to its receptor activates three major signalling pathways in prostate cells: the Janus tyrosine family kinase (JAK)-signal transducer and activator of transcription (STAT) pathway, the ERK1/2-MAPK pathway, and the PI3K pathway [365–370]. These signalling cascades regulate an array of survival mechanisms, most notably inhibiting apoptosis and stimulating proliferation and EMT [365–370].

Similar findings have been found for IL-8, whose expression levels correlate with PCa aggressiveness [371–373]. The intricate signalling network initiated by IL-8 upon binding to its receptors, CXCR1 and CXCR2, was shown to activate PI3K, AKT, MAPK, and phospholipase C, triggering downstream crosstalk of PI3K with JAK/STAT3 [374–376]. The reported cellular processes affected in response included cell proliferation, invasion, migration, survival, metabolism, and angiogenesis [374–377]. Interestingly, IL-8 signalling has been associated with the transcriptional activity of AR, implicating it in the transition from androgen-sensitive to androgen-insensitive PCa, as well as in radiation and chemotherapeutic resistance [378–380]. Beyond the direct impact on tumour cells, CXCR2-signalling is important in angiogenesis and in infiltrating immune cells, namely neutrophils and TAMs, suggesting that IL-8 may have a significant pro-angiogenic and tumorigenic role within the TME [373, 380–383]. Despite being less studied, the association between IL-22/IL-1β signalling and PCa has also been demonstrated [384, 385].

Considering TNF-α, a type II transmembrane protein with an intracellular N terminus and signalling potential both as a membrane-integrated protein and a soluble cytokine released after proteolytic cleavage [386, 387], the activation of its receptor (TNFR) led to the recruitment of intracellular adaptor proteins that activate multiple signal transduction pathways [387, 388]. TNFR1 activation can induce a range of inflammatory mediators and growth factors via the activation of the AP-1 transcription factors or IKβ kinases, which, in turn, activate NF-κB [388]. In the TME, TNF-α can act as an endogenous tumour promoter [389]. In the context of PCa, Rodríguez-Berriguete et al. demonstrated that increased tumour expression of TNF-α and TNFR1 is significantly correlated with poor prognosis [390]. Moreover, significantly elevated serum levels of TNF-α were found in patients with metastatic disease compared to those with localised tumours (6.3 ± 3.6 vs. 1.1 ± 0.5 pg/mL, respectively) [362]. This pattern was also observed for MCP-1, where elevated levels were observed in patients with bone metastases compared to individuals with localised PCa [391, 392]. Moreover, augmented levels in subjects starting ADT were associated with shorter times to the development of castration-resistance and overall survival [391, 392]. MCP-1 receptor (CCR2) expression also positively correlates with Gleason score and clinical pathological stages [393]. MCP-1 is a member of the C–C motif chemokine superfamily that plays a critical role in the recruitment and activation of monocytes during inflammation and angiogenesis. MCP-1 facilitates monocyte recruitment, angiogenesis, and, notably, PCa (LNCaP and PC3) cell invasion, especially through MMP-2 activation [394–397]. Knockdown of CCR2 or MCP-1 on C4-2B and PC3 neoplastic prostate cells significantly diminished invasion and tumour cell-induced osteoclast activity [391], which is of paramount relevance for the development of PCa metastasis. Indeed, this study also showed that MCP-1 knockdown diminished PC3 xenograft growth in bone, demonstrating its importance in the metastatic process [391].

Another C–C motif chemokine superfamily member target of obesogens is CCL20. It was shown to induce the proliferation of PCa cells, both directly through CCR6-mediated proliferation and indirectly through enhanced recruitment of inflammatory cells such as Th17 [398]. Immunofluorescence analysis surprisingly showed that the PCa cells themselves, when adjacent to inflammation, stained prominently for CCL20, and an analysis of a 40-specimen PCa array supported the relationship between CCL20 expression and cancer-associated inflammation [398]. Analysis of these PCa specimens also showed a significant correlation between CCL20 expression and both high-stage (*p* < 0.05 for expression of CCL20 in Stage IV vs. Stage II) and high-grade (*p* = 0.02 for expression of CCL20 in cases with Gleason scores 8–10 vs. 6–7) PCa [398].

Despite the substantial body of evidence, much remains to be understood regarding the obesogenic dysregulation of C–C motif chemokine signalling impacting PCa progression.

## Potential Druggable Targets to Mitigate the Effects of Obesogens

The previously described effects of obesogens in earlier chapters of this review, along with the underlying mechanisms of action, point to a set of potential druggable targets (Table 3 ) that could be used to mitigate the damage induced by obesogens in PCa.

**Table 3:** Potential targets and drugs to mitigate the effects of obesogens in prostate cancer

Biologic process	Potential therapeutic approach	Drug	Refs
Adipogenesis and lipid metabolism	PPAR-γ antagonists	GW9662 T0070907 Imatinib Hit small molecules (*e.g.* NCS 404243 and 400,401*)*	[399–402]
Inflammation	Anti-inflammatory agents	Next-generation probiotics	[403]
Endocrine pathway disruption	Endocrine modulators	ICI 182,780	[404]
Epigenetic modifications	Inhibitors of BET family proteins	ABBV-744	[406]

*BET* Bromodomain and extra-terminal, *PPAR-γ* Peroxisome proliferator-activated receptor gamma.

The activation of PPAR-γ is a common denominator and a downstream target in the action of several obesogens [104, 124, 152, 207, 217], representing a putative approach for therapeutic intervention. PPAR-γ antagonists, such as T0070907 and GW9662, have been demonstrated to interfere with cancer cell metabolism, apoptosis, and migration, enhancing the efficacy of anticancer drugs [399, 400]. Within this interplay, imatinib, a tyrosine-kinase inhibitor used to treat several human malignancies, also demonstrated beneficial effects on both glucose/lipid metabolism and energy homeostasis by blocking PPAR-γ phosphorylation, increasing the browning of WAT and energy expenditure [401]. Of note, a study identified a panoply of hit small molecules (allosteric and orthosteric binders) capable of inhibiting the PPAR-γ activity in PCa cells, underscoring the importance of future investigations exploiting their potential as anti-cancer agents [402].

Recently, research has also focused on identifying and culturing specific microbiota taxa that can counteract the pro-inflammatory effects of obesogens, known as next-generation probiotics [403]. Specifically, a promising avenue of research is introduced by the demonstration that the pathophysiology of inflammation exacerbated by the obesogen BPA could be modified by tolerant bacterial species with anti-inflammatory properties [403]. These probiotics would potentially repair the gut microbiota disrupted by obesogens, thereby mitigating obesity and related metabolic disorders [403].

Endocrine-disrupting potential is a classic hallmark underlying the obesogenic actions of EDC, which can also be targeted for therapeutic strategies. In this regard, a study demonstrated that co-treatment with the ER inhibitor ICI 182,780 significantly inhibited BPF-induced lipid accumulation in ASCs [404], suggesting that selective modulation of ER–dependent pathways may represent an effective approach to counteract the adipogenic and metabolic consequences of exposure to bisphenol analogues.

Obesogens are also known to induce epigenetic changes that affect the expression of genes related to the control of adipogenesis, metabolism, and energy balance [104, 251, 405]. Proteins of the bromodomain and extra-terminal (BET) domain family are epigenetic readers that bind acetylated histones through their bromodomains to regulate gene transcription [406]. ABBV-744, a highly potent and selective inhibitor of the bromodomain 2 of BET family proteins with drug-like properties, demonstrated anti-proliferative effects on PCa cells, underscoring its relevance as a potential therapeutic target to mitigate the impact of obesogens [406]. Table 3 summarises the information on pharmacologic interventions regarding lipid metabolism, adipose tissue inflammation, epigenetic modifications and endocrine signalling pathways.

## Conclusion and Future Directions

Several obesogens have been identified to date, encompassing a panoply of ubiquitous compounds in everyday life activities. Although their mechanisms of action are diverse, they converge on common outcomes: dysregulation of adipocytes and body weight gain. Therefore, obesogens have been considered a significant contributing factor to the global obesity epidemic.

As described in the present review, obesogens encompass distinct classes of compounds, including organotins (TBT, TPT and metabolites), pesticides (OCs, OPs, carbamates, or pyrethroids, as well as neonicotinoids), plasticisers (BPA and substitutes, phthalates), food/cosmetic/pharmaceutical additives (emulsifiers, preservatives, parabens, MSG), and flame retardants (PBDEs, OPFRs). Regardless the type of compound, obesogenic effects are mainly mediated through activation of nuclear receptors (especially PPAR-γ and RXR), disruption of lipid and glucose metabolism, adipocyte differentiation, inflammation, and altered appetite or satiety regulation. Notably, many of these compounds show effects even at low or nanomolar concentrations, which are often more pronounced in obesity conditions. They also exhibit persistence, bioaccumulation in adipose tissue, and in some cases sex-dependent responses, highlighting the complexity of management and establishing security thresholds for obesogen exposure. Moreover, several obesogens demonstrate transgenerational and epigenetic effects, with prenatal or perinatal exposures linked to obesity and metabolic dysfunction in later generations, underscoring the long-term risk of these persistent chemicals to human health. Notwithstanding the reported obesogenic effects of such a diverse range of compounds, the molecular mechanisms underlying their actions remain largely unexplored. Additionally, there is an urgent need for comprehensive biomonitoring studies to identify other emerging obesogenic compounds, evaluate their environmental prevalence and persistence, and assess their impact on human health.

As discussed, the role of obesogens in the relationship with PCa remains insufficiently understood. Nevertheless, existing evidence suggests that these chemicals may contribute to the development and progression of PCa through their dual role in disrupting hormonal and metabolic pathways, by direct actions in prostate cells or via induced dysregulation of adipose tissue. At the cellular level, obesogens modulate AR and ER signalling, disrupt epigenetic regulators, and trigger proliferative, invasive, and survival pathways in PCa cells, facilitating tumour progression to more aggressive phenotypes. Beyond the direct actions in the prostate epithelium, obesogens induce profound alterations in adipose tissue, promoting the impairment of lipid homeostasis, hyperleptinemia, and chronic low-grade inflammation. These changes generate a systemic and local milieu rich in metabolic substrates, hormones, and proinflammatory cytokines that fuel tumour growth. Interestingly, ASCs have been recognised as critical mediators of PCa progression [33, 407]. These findings, together with mechanistic studies showing that certain environmental toxicants can dysregulate ASCs function and promote pro-tumoral signalling [408], emphasise the need to keep exploring how obesogens affect ASCs biology, investigating the potential contribution of this dysregulation to prostate carcinogenesis. Overall, further research is needed to clarify the liaison between obesogens, altered adipose tissue and PCa. However, the available data suggest that obesogens-dysregulated adipose tissue acquires an obesity-like phenotype characterised by excessive adiposity, impaired metabolic function and a proinflammatory status, which might play a crucial role in altering prostate cell fate, being reasonable to assume that the procarcinogenic effects of obesogens are mainly primed by the adipose tissue.

**Fig. 4:**
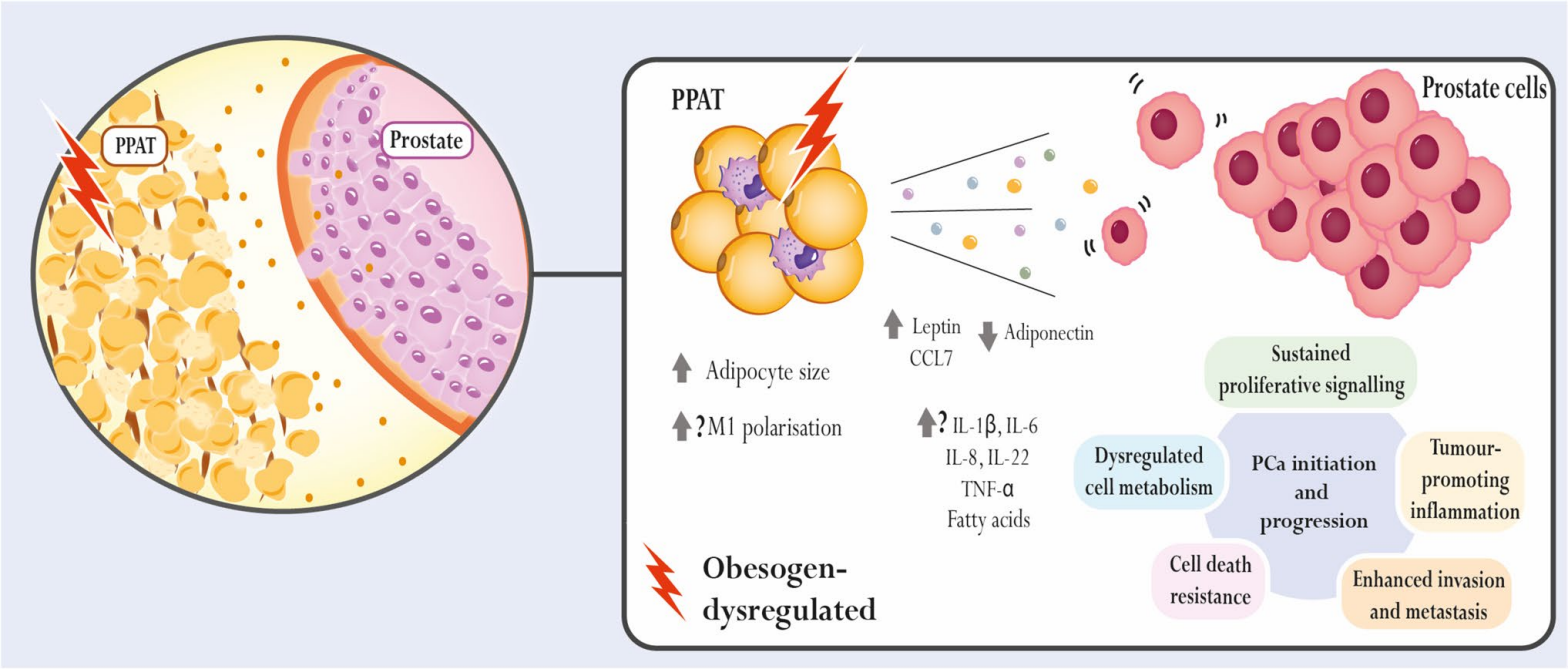
Hypothesis model of obesogen-dysregulated periprostatic adipose tissue (PPAT)-driven prostate cancer (PCa) development. PPAT is a target of obesogenic dysregulation [355], which alters its phenotype and secretome. Exposure to obesogens increases adipocyte size in PPAT, with possible polarisation of the macrophage population towards the M1 stage. PPAT secretome shaped by obesogens is characterised by the increased levels of leptin, whereas adiponectin is decreased. Moreover, obesogenic exposure increases the levels of the C–C motif chemokine ligand 7 (CCL7) as well as might increase the levels of pro-inflammatory interleukins (IL) and tumour necrosis factor a (TNF-a). Higher levels of fatty acids detected in the secretome of obesogen-exposed adipose tissue are also plausible to be observed in the PPAT secretome. Overall, obesogen-dysregulated PPAT plays a critical role in PCa initiation and progression, driving survival and migration of prostate cells. Legend: ↑: increase; ↓: decrease;?: alterations observed in white fat depots to be confirmed in PPAT

Given the anatomical intimacy between the prostate and its surrounding PPAT, over the past decade, attention has focused on the role of PPAT in PCa onset and progression. The great majority of studies to date describe the tumour–promoting effects driven by PPAT, which lead to increased proliferation and invasion of PCa cells, fuelling cancer [318–322]. Nevertheless, studies exist reporting no effect or even a tumour-suppressive role of the PPAT [317, 409–411]. These seemingly contradictory observations likely reflect PPAT depot heterogeneity or methodological differences among studies. This duality of effects highlights the importance of characterising histologically and biochemically the PPAT in framing future research efforts and the need to fully clarify the factors that induce PPAT remodelling, shifting its condition towards a tumour-promoting phenotype. Notably, evidence for a neutral or tumour-suppressive role of PPAT comes from non-obese individuals or low-grade PCa patients [317, 411], suggesting that the PPAT–prostate communication becomes particularly relevant in obesity and aggressive PCa. Globally, the available literature supports the notion that obesogen-induced PPAT dysfunction emerges as a critical mediator of PCa aggressiveness, as the "obese" PPAT has demonstrated an enhanced potential to drive PCa development [312–316]. Adipose depot-specific responses under obesogenic dysregulation are crucial to underscore, as it is highly likely that obesogenic effects differ across distinct types of adipose depots. To the best of our knowledge, only one study to date has directly examined the impact of obesogens on PPAT. Very recently, we demonstrated that TBT can dysregulate PPAT, significantly altering its secretome and impacting prostate cell viability, survival and migration features [355]. This review further highlights the importance of considering PPAT-prostate interorgan communication as a critical component in prostate carcinogenesis, particularly given the key role that the PPAT can have in modulating the TME towards fuelling cancer development, which can be exacerbated by the influence of obesogenic EDCs Fig. 4.

A significant knowledge gap persists regarding the mechanistic pathways linking obesogen exposure, PPAT remodelling, and PCa progression. Understanding how obesogenic EDCs modulate the PPAT secretome and its immune profile and how these changes influence prostate cell behaviour could reveal new molecular targets and inform preventive strategies. Unravelling this complex crosstalk will be crucial for identifying novel therapeutic opportunities and preventing progression to aggressive or metastatic forms of PCa, especially in obese patients. Future research should prioritise mechanistic studies that integrate endocrine, metabolic, and immunological pathways to unravel how obesogens converge on the adipose tissue-prostate axis to fuel tumour initiation and progression. Special emphasis should be given to investigating the effects of long-term and low-dose exposures, as well as the transgenerational effects, and the cumulative impact of complex chemical mixtures, as these better reflect real-life scenarios. Advancing *in vivo* models that capture the interplay between prostate cells, PPAT, and systemic metabolic cues will be crucial for translating findings into human-relevant contexts and confirming the usefulness of the identified potential druggable targets Table 3. At present, no established pharmacological therapies are available to counteract obesogen-induced endocrine and metabolic dysregulation in PCa. However, emerging evidence suggests that targeting adipose tissue inflammation, lipid metabolism and hormone signalling pathways may represent promising avenues for future therapeutic development, including pharmacological approaches using PPAR-γ and ER antagonists, probiotics and epigenetic regulators [403, 404]. Further research toward the development of comprehensive and effective therapeutic strategies is therefore essential. In parallel, strengthening the regulatory frameworks that could limit exposure to known or suspected obesogens will be pivotal. Public health initiatives should also aim to raise awareness about the potential risks for cancer development associated with obesogen exposure and promote lifestyle choices that minimise exposure (*e.g.*, reducing the consumption of processed food, minimising plastic use, etc.).

## Key References

- Harb AA, Shechter A, Koch PA, St-Onge M-PJ. Ultra-processed foods and the development of obesity in adults. Eur J Clin Nutr. 2023; 77 (6):619-27; 10.1038/s41430-022-01225-z
  - The presented evidence supports a positive relationship between high ultra-processed foods and obesity, highlighting the need for future investigations to establish causality and elucidate mechanisms.

- Kladnicka I, Bludovska M, Plavinova I, Muller L, Mullerova D. Obesogens in foods. Biomolecules. 2022; 12(5):680; 10.3390/biom12050680
  - This review highlights that although some obesogens occur naturally in food, most are introduced as contaminants or additives and are particularly abundant in ultra-processed foods.

- Li H, Li F, Zhou C, Bu J, Yang H, Zhong L, et al. Exposure to OPFRs is associated with obesity and dysregulated serum lipid profiles: data from 2017–2018 NHANES. Metabolites. 2024; 14(2):124; 10.3390/metabo14020124
  - The authors explored the association between the concentrations of organophosphorus flame retardants (OPFR) metabolites, body mass index, obesity, and serum lipid profiles, concluding that environmental exposure to this type of compound might contribute to obesity and dysregulate lipid homeostasis in adults.

- Stratakis N, Rock S, La Merrill MA, Saez M, Robinson O, Fecht D, et al. Prenatal exposure to persistent organic pollutants and childhood obesity: A systematic review and meta‐analysis of human studies. Obes Rev. 2022; 23:e13383; 10.1111/obr.13383
  - Epidemiological studies suggest that prenatal exposure to persistent organic pollutants is associated with greater adiposity in childhood.

- Sousa S, Rede D, Fernandes VC, Pestana D, Faria G, Delerue-Matos C, et al. Accumulation of organophosphorus pollutants in adipose tissue of obese women-metabolic alterations. Environ Res. 2023;239:117337; 10.1016/j.envres.2023.117337
  - Organophosphorus pollutants were detected in the adipose tissue of obese women, demonstrating not only the ability of these compounds to accumulate in adipose depots, but also that anthropometric and hormonal parameters, such as cholesterol, glycaemia, macrominerals, urea and sedimentation velocity, might be influenced by their presence.

- Zhang D, Zhao K, Han T, Zhang X, Xu X, Liu Z, et al. Bisphenol A promote the cell proliferation and invasion ability of prostate cancer cells via regulating the androgen receptor. Ecotoxicol Environ Saf. 2024;269:115818; 10.1016/j.ecoenv.2023.115818
  - Leveraging online datasets enabled the identification of associations between exposure to the obesogen bisphenol A and prostate cancer. Moreover, it was demonstrated that exposure to bisphenol A could markedly augment the proliferative and invasive capabilities of prostate cancer cells, corroborating the need for deeper exploration into the mechanistic links between obesogenic EDCs exposure and prostate cancer progression.

## Data Availability

No datasets were generated or analysed during the current study.
